# A genetic approach to identify amino acids in Gcn1 required for Gcn2 activation

**DOI:** 10.1371/journal.pone.0277648

**Published:** 2022-11-28

**Authors:** Susanne Gottfried, Siaosi M. B. M. J. Koloamatangi, Clement Daube, Anja H. Schiemann, Evelyn Sattlegger

**Affiliations:** 1 School of Natural Sciences, Massey University, Auckland, New Zealand; 2 School of Natural Sciences, Massey University, Palmerston North, New Zealand; 3 Maurice Wilkins Centre for Molecular BioDiscovery, Massey University, Palmerston North, New Zealand; Tulane University Health Sciences Center, UNITED STATES

## Abstract

The protein kinase Gcn2 is present in virtually all eukaryotic cells. It is best known for its role in helping cells cope with amino acid starvation. Under starvation, Gcn2 phosphorylates the α subunit of the eukaryotic translation initiation factor 2 (eIF2α), to stimulate a signal transduction pathway that allows cells to cope and overcome starvation. Gcn2 has been implicated in many additional biological functions. It appears that for all functions, Gcn2 must directly bind to its effector protein Gcn1, mediated via a region in Gcn1 called the RWD binding domain (RWDBD). Arg-2259 in this region is important for Gcn2 binding. Overexpression of a Gcn1 fragment only encompassing the RWDBD binds Gcn2, thereby disrupting endogenous Gcn1-Gcn2 interaction which dampens Gcn2 activation. Consequently, cells are unable to increase eIF2α phosphorylation under starvation conditions, visible by impaired growth. This dominant negative phenotype is reverted by the R2259A substitution, again allowing Gcn1-Gcn2 interaction and enhanced eIF2α phosphorylation. We have found that the amino acid substitutions, R2289A, R2297A, and K2301A, also reverted the dominant negative phenotype as well as allowed enhanced eIF2α phosphorylation, as found previously for the R2259A substitution. This suggests that the respective amino acids are relevant for the overexpressed RWDBD to disrupt Gcn1-Gcn2 interaction and impair Gcn2 activation, supporting the idea that in Gcn1 these amino acids mediate Gcn2-binding. Our findings suggest that two helices in Gcn1 constitute a Gcn2 binding site. We serendipitously found amino acid substitutions that enhanced the dominant negative phenotype that correlated with a further reduction in eIF2α-P levels, suggesting that the respective RWDBD variants are more potent in disrupting Gcn1-Gcn2 interaction.

## Introduction

The protein kinase General control non-derepressible 2 (Gcn2) is part of a signal transduction pathway present in virtually all eukaryotic cells, that adjusts protein synthesis to the cell’s needs [1]. This pathway has been best studied in the yeast *Saccharomyces cerevisiae*, where it was first found that starvation for a single amino acid leads to the synthesis of virtually all 20 amino acids. For this reason, this pathway was called General Amino Acid Control (GAAC) pathway in yeast.

Studies suggest that, in response to detecting amino acid shortage, Gcn2 phosphorylates the alpha subunit of eukaryotic translation initiation factor 2 (eIF2α). This leads to reduced global protein synthesis (though this likely only occurs under severe starvation conditions elicited under experimental conditions in the laboratory). Most importantly, eIF2α phosphorylation leads to the increased translation of transcriptional activators, Gcn4 in yeast and ATF4 in mammals, subsequently triggering a change in the cell’s gene expression profile to allow cells to adapt to and overcome the adverse condition [2, 3]. While yeast only harbours Gcn2, mammals possess three additional eIF2α protein kinases, each responding to specific stress conditions, hence this system was named the integrated stress response in mammals [1].

The exact mechanism by which Gcn2 detects starvation is still not fully understood. The first working model proposed that Gcn2 must bind directly to its effector protein Gcn1, and both must bind to the ribosome [4, 5]. In analogy to the bacterial system, this model proposed that under starvation conditions, when the cognate charged amino acid is not available, an uncharged tRNA enters the A-site in a codon specific manner. This tRNA is then transferred to the Gcn2 Histidyl-tRNA synthesis-like domain, subsequently leading to intramolecular re-arrangements of Gcn2 to stimulate its kinase domain, which also involves Gcn2 auto-phosphorylation [3]. Activated Gcn2 then phosphorylates its substate eIF2α. Recent findings support another model in which ribosomal stalk proteins are involved in mediating Gcn2 activation [6–8]. The link between uncharged tRNAs and the P-stalk remains to be determined in view of Gcn2 activation under amino acid starvation in yeast and mammals. No matter the mechanism of Gcn2 activation, studies strongly suggest that Gcn1, and direct Gcn1-Gcn2 interaction, are essential for Gcn2 activation in yeast as well as mammals, and so are ribosome-Gcn1 and ribosome-Gcn2 interactions [5, 9, 10]. Supporting this finding, it has recently been shown that deletion of Gcn1 in mice abolishes Gcn2 activation [11].

Gcn2 has been implicated in more functions than maintaining amino acid homeostasis, such as coping with oxidative stress, long-term memory formation, feeding behaviour and immune system regulation [1]. Studies so far suggest that Gcn1 is essential for all of the biological roles executed by Gcn2 [1]. Furthermore, proteins have been found that disrupt Gcn1-Gcn2 interaction as a way of regulating Gcn2 activity in yeast as well as mammals [12–18], underscoring the biological importance of Gcn1-Gcn2 interaction for finetuning Gcn2 activation in a spatiotemporal manner [1].

The direct Gcn1-Gcn2 interaction has not been thoroughly investigated. So far, a region in Gcn1 was identified that encompasses the Gcn2-binding site, spanning Gcn1 amino acids 2052–2428, that is necessary and sufficient for Gcn2-binding *in vivo* and *in vitro* [5]. A parallel study showed that the Gcn1 fragment 2048–2382 interacted with Gcn2 in overlay and yeast-two-hybrid assays [19]. Overexpression of either fragment in a wild-type yeast strain impaired growth under starvation conditions, i.e. the fragment causes a dominant negative phenotype. Since Gcn2 activation is necessary for cells to overcome starvation and grow, this observed phenotype aligns with the idea that Gcn2 activation was impaired [5, 19]. We then provided several lines of evidence that disruption of Gcn1-Gcn2 interaction did not affect other known Gcn1 functions, such as ribosome binding and binding to Gcn20, suggesting that impaired Gcn1-Gcn2 interaction *per se* is the cause of impaired Gcn2 activation [5].

The 2052–2428 Gcn1 fragment co-precipitates *in vitro* with the Gcn2 N-terminal 598 amino acids containing the RWD domain (a domain found in RING finger-containing proteins, WD-repeat-containing proteins, and yeast DEAD (DEXD)-like helicases) [5]. Therefore, for simplicity this Gcn1 fragment was called the RWD binding domain (RWDBD) [20]. Though it shall be noted that the RWDBD does not necessarily constitute an autonomous structural domain. Overexpressed RWDBD co-precipitates Gcn2 *in vivo*, and this correlates with reduced Gcn1-Gcn2 interaction, providing evidence that the RWDBD competes with Gcn1 for Gcn2 binding [5]. We have shown that overexpression of the equivalent mouse fragment (amino acids 2204–2651) abolishes Gcn2 activation [17], suggesting that Gcn1-Gcn2 interaction is conserved from yeast to mammals.

The exact Gcn2 binding site has not been revealed yet in the RWDBD of Gcn1. Thus far, only one amino acid in the RWDBD, Arg-2259, was identified as critically required for direct Gcn1-Gcn2 binding *in vivo* and *in vitro* [5]. R2259A substitution in full-length Gcn1 dramatically reduces Gcn1-Gcn2 interaction *in vivo*, and this correlates with reduced growth under starvation conditions. Furthermore, R2259A substitution in the RWDBD fragment drastically impairs its dominant negative effect. However, past findings suggest that Arg-2259 is not the only amino acid in Gcn1 that mediates Gcn2 binding. For example, *gcn1-R2259A* yeast cells show improved growth under starvation conditions when Gcn2 was overexpressed, but *gcn1Δ* cells do not. This suggests that Gcn1-R2259A still has some affinity to Gcn2. Increased Gcn2 levels could compensate—by mass action—for the reduced Gcn2-affinity of Gcn1-R2259A, thereby allowing again some Gcn1-Gcn2 interaction and concomitant Gcn2 activation. Hence, this indicates that there must be amino acids besides Arg-2259 that are relevant for Gcn1-Gcn2 interaction.

Given the relevance of Gcn1 in Gcn2 activation, and the fact that other RWD domain containing proteins modulate Gcn2 activity by competing with Gcn2 for Gcn1 binding [12–18], it is of high importance to better understand which parameters in Gcn1 contribute to Gcn2 binding. The structure of the Gcn1 RWDBD has been modelled computationally [20]. The RWDBD contains several HEAT repeats, where each repeat contains two helixes separated by an unstructured region. The first helices of each repeat pack next to each other in parallel to form one surface-side of the protein, while the second helices of each repeat also pack next to each other in parallel to form the second layer of helices to form the other surface-side of the protein. According to our RWDBD structure model, Arg-2259 is surface exposed and situated at the end of one helix [5, 20]. Arg-2259 is located amongst similarly charged amino acids, which are surrounded by an uncharged region, suggesting that the charged amino acids are contributing to Gcn2 binding. From this model we chose candidate amino acids that may contact Gcn2, as well as additional amino acids as controls. These were individually subjected to amino acid substitutions in the RWDBD. We then tested whether these substitutions revert the dominant negative effect elicited by the overexpressed RWDBD. In fact, we found three amino acid substitutions that each reverted the dominant negative effect elicited by the RWDBD, and this correlated with improved levels of eIF2α phosphorylation, as found previously by the R22259A substitution [5]. This suggests that the respective amino acids are relevant for Gcn1-Gcn2 interaction. Curiously, we also found amino acid substitutions that enhanced the dominant negative phenotype, and this correlated with a further reduction in eIF2α-P levels, suggesting that the respective RWDBD variants are more potent in disrupting Gcn1-Gcn2 interaction.

## Methods

### Strains and plasmids

The yeast strains and plasmids used in this study are summarised in Tables 1 and 2. The vectors used were YEp13 [21], pRS316 [22], pEG(KT) and the same plasmid but deleted for the *leu2d* marker, pES128-9 [5].

**Table 1:** Strains used in this study.

strain	Genotype	source
**Genetic background H1511**		
H1511	*MATα ura3-52 trp1-63 leu2-3*,*112*, *GAL2*^*+*^	[32]
H2556	same as H1511 but *gcn1Δ*	[5]

**Table 2:** Plasmids used in this study.

plasmid	gene	selectable marker	vector	source
*Yeast gene fusions*, *expressing Gcn1 RWDBD (amino acids 2052–2428) from a Galactose inducible promotor*				
pES124-B1	*GST* ^a^ *-RWDBD*	*Amp*^*R*^, *URA3*, *leu2d*	pEG(KT), 2μ	[22]
pES125-B2-1	*GST* ^a^ *-RWDBD*	*Amp*^*R*^, *URA3*, *leu2Δ*	pES128-9, 2μ	[5]
pES167-2E	*GST* ^a^ *-RWDBD-R2259A*	*Amp*^*R*^, *URA3*, *leu2d*	pEG(KT), 2μ	[5]
pESY13223	*GST* ^a^ *-RWDBD-N2224A-myc* ^b^	*Amp*^*R*^, *URA3*, *leu2d*	pEG(KT), 2μ	this work
pESY13224	*GST* ^a^ *-RWDBD-R2227A-myc* ^b^	*Amp*^*R*^, *URA3*, *leu2d*	pEG(KT), 2μ	this work
pESY13225	*GST* ^a^ *-RWDBD-N2245A-myc* ^b^	*Amp*^*R*^, *URA3*, *leu2d*	pEG(KT), 2μ	this work
pESY13226	*GST* ^a^ *-RWDBD-K2247A-myc* ^b^	*Amp*^*R*^, *URA3*, *leu2d*	pEG(KT), 2μ	this work
pESY13227	*GST* ^a^ *-RWDBD-S2251A-myc* ^b^	*Amp*^*R*^, *URA3*, *leu2d*	pEG(KT), 2μ	this work
pESY13228	*GST* ^a^ *-RWDBD-V2261D-myc* ^b^	*Amp*^*R*^, *URA3*, *leu2d*	pEG(KT), 2μ	this work
pESY13229	*GST* ^a^ *-RWDBD-V2261A-myc* ^b^	*Amp*^*R*^, *URA3*, *leu2d*	pEG(KT), 2μ	this work
pESY13244	*GST* ^a^ *-RWDBD-E2263A-myc* ^b^	*Amp*^*R*^, *URA3*, *leu2d*	pEG(KT), 2μ	this work
pESY13245	*GST* ^a^ *-RWDBD-R2264A-myc* ^b^	*Amp*^*R*^, *URA3*, *leu2d*	pEG(KT), 2μ	this work
pESY13230	*GST* ^a^ *-RWDBD-K2270A-myc* ^b^	*Amp*^*R*^, *URA3*, *leu2d*	pEG(KT), 2μ	this work
pESY13231	*GST* ^a^ *-RWDBD-R2289A-myc* ^b^	*Amp*^*R*^, *URA3*, *leu2d*	pEG(KT), 2μ	this work
pESY13232	*GST* ^a^ *-RWDBD-F2291E-myc* ^b^	*Amp*^*R*^, *URA3*, *leu2d*	pEG(KT), 2μ	this work
pESY13233	*GST* ^a^ *-RWDBD-Q2294A-myc* ^b^	*Amp*^*R*^, *URA3*, *leu2d*	pEG(KT), 2μ	this work
pESY13234	*GST* ^a^ *-RWDBD-Q2294D-myc* ^b^	*Amp*^*R*^, *URA3*, *leu2d*	pEG(KT), 2μ	this work
pESY13235	*GST* ^a^ *-RWDBD-R2297A-myc* ^b^	*Amp*^*R*^, *URA3*, *leu2d*	pEG(KT), 2μ	this work
pESY13236	*GST* ^a^ *-RWDBD-K2301A-myc* ^b^	*Amp*^*R*^, *URA3*, *leu2d*	pEG(KT), 2μ	this work
pESY13237	*GST* ^a^ *-RWDBD-D2305A-myc* ^b^	*Amp*^*R*^, *URA3*, *leu2d*	pEG(KT), 2μ	this work
pESY13238	*GST* ^a^ *-RWDBD-E2309A-myc* ^b^	*Amp*^*R*^, *URA3*, *leu2d*	pEG(KT), 2μ	this work
pESY13239	*GST* ^a^ *-RWDBD-R2328A-myc* ^b^	*Amp*^*R*^, *URA3*, *leu2d*	pEG(KT), 2μ	this work
pESY13240	*GST* ^a^ *-RWDBD-D2330A-myc* ^b^	*Amp*^*R*^, *URA3*, *leu2d*	pEG(KT), 2μ	this work
pESY13241	*GST* ^a^ *-RWDBD-E2335A-myc* ^b^	*Amp*^*R*^, *URA3*, *leu2d*	pEG(KT), 2μ	this work
pSG07	*GST*^a^*-RWDBD-E1-myc*^b^, containing substitutions *K2247A*, *V2261A*, *E2263A*, *R2264A*, *Q2294A*			
		*Amp*^*R*^, *URA3*, *leu2Δ*	pES128-9, 2μ	this work
pSG08	*GST*^a^*-RWDBD-AV1-myc*^b^, containing substitutions R2259A, K2270A, R2289A, R2297A, K2301A			
		*Amp*^*R*^, *URA3*, *leu2Δ*	pES128-9, 2μ	this work
pSG09	*GST*^a^*-RWDBD-AV2-myc*^b^ containing substitutions R2259A, R2289A, R2297A, K2301A			
		*Amp*^*R*^, *URA3*, *leu2Δ*	pES128-9, 2μ	this work
pSG10	*GST*^a^*-RWDBD-AV3-myc*^b^ containing substitutions R2259A, K2270A, R2297A, K2301A			
		*Amp*^*R*^, *URA3*, *leu2Δ*	pES128-9, 2μ	this work
pAH15	*Gcn2*	*Amp*^*R*^, *Leu2*	Yep13, 2μ	[33]
p2367	*GCN1-myc* ^b^	*Amp*^*R*^, *URA3*	pRS316	[25]
pES174-3-2	*Gcn1-R2259A-myc* ^b^	*Amp*^*R*^, *URA3*	pRS316	[5]
pAS23	*Gcn1-AV2-myc* ^b^	*Amp*^*R*^, *URA3*	pRS316	this work

*a* epitope tag at the N-terminus of the ORF

*b* epitope tag at the C-terminus of the ORF

Plasmids expressing mutant RWDBD from a galactose inducible promotor were generated as described elsewhere [23]. Briefly, in pES124-B2 or pES125-B2-1, the C-terminal portion of the RWDBD—covering the area of the to-be mutated amino acids—was removed using *Hind*III which cuts twice in the RWDBD and C-terminal to the RWDBD in the multiple cloning site of the plasmid. Using the uncut pES124-B2 as template, the same C-terminal portion of the RWDBD—but containing the desired amino acid substitution(s)—was generated via fusion PCR, using primers to integrate the mutation, and outside primers that also integrated a myc-tag at the C-terminus of the RWDBD, following standard procedures [24]. The PCR amplicon was then subjected to an extension PCR using primers that anneal to the ends of the PCR amplicon, and that harboured a 60 bases long 5’ end that was homologous to the bases at either end of the linearised plasmid. The linearised plasmid and the final PCR amplicon were transformed into yeast to generate the circular plasmid via in-yeast recombination [24]. All constructs were verified via commercial sanger sequencing.

Plasmid pAS23-flGcn1-AV2 expressing Gcn1-AV2-myc from its native promotor, was generated by replacing the *Blp*I-*Age*I fragment in plasmid p2367 [25] by a commercially synthesised *Blp*I-*Age*I fragment coding for the same amino acids except of the AV2 amino acid substitutions, followed by sequence verification (Genscript).

### Semi-quantitative growth assays

These assays were performed as described in [23]. Briefly, ten-fold serial dilutions of saturated overnight cultures were generated using synthetic medium lacking a carbon source. 5 μL of each dilution was transferred onto solid medium containing the necessary supplements to cover auxotrophies, glucose or galactose as carbon source, and additional supplements if indicated. Plates were incubated at 30°C, and the growth documented with a document scanner. Each plate contained, as a reference, transformants expressing GST alone and un-mutated RWDBD, respectively.

Cell growth was quantified as follows. For each strain on a plate, for each dilution a score for growth was given from 0 to 10, with score 10 being full growth, and the sum of the scores determined (total score). Then for each strain, the total score on the plate containing both galactose and 3-amino-1,3,4-triazole (3AT) (the 3AT containing plate showing the largest growth difference was chosen) was divided by the score on the plate containing galactose alone, leading to an adjusted growth score. In order to compare the growth of all strains across all semi-quantitative growth assays, and in order to better illustrate growth differences, we next aimed to set the growth of strains with impaired Gcn2 function to ‘zero’ (inhibition of Gcn2 activity by the overexpressed un-mutated RWDBD), and to set the growth of strains with fully functional Gcn2 to ‘one’ (strains overexpressing GST alone). To do this, for each semi-quantitative growth assay, we subtracted the average adjusted growth score of the strains overexpressing un-mutated RWDBD, from the adjusted growth score of each of the other strains (meaning that the average growth score of the strains overexpressing un-mutated RWDBD was set to ‘zero’). Next, the resulting scores were divided by that of the strain overexpressing GST, leading to relative growth scores where that of the strain overexpressing GST was set to ‘one’.

### Generating cell extracts for immunoblotting

Yeast cell extracts were generated as published previously [26]. Briefly, cells were grown to exponential phase in liquid minimal medium. Cells were exposed to formaldehyde before harvesting, and then cell pellets were subjected to cell lysis to generate cell extracts. To score for eIF2α-P levels, the same was done as above, just that the exponentially growing cells were exposed to 3AT to elicit starvation, prior to harvesting [26].

### Immunoblotting

Cell extracts were resolved via SDS polyacrylamide gel electrophoresis (SDS-PAGE) using 4–20% gradient gels, the proteins were transferred onto a nitrocellulose membrane, and immunoblotting was carried out using rabbit polyclonal antibodies against phosphorylated eIF2α (1:2000, 44728G; Life Technologies), GST (1:5000; Santa Cruz, SC-459), and mouse monoclonal antibody against Pgk1 (1:5000, 459250; Life Technologies) and the myc epitope tag (1:500, Santa Cruz, SC-40). The primary antibodies were detected with HRP-linked secondary anti-rabbit antibodies (1:100000; Pierce, Rockford, IL, USA) or anti-mouse antibodies (1:50000; Pierce). The proteins were visualised by chemiluminescence using the LAS 4000 imager (GE Healthcare Life Sciences, Chicago, IL, USA). The intensity of signals was quantified using the IMAGE J software [27]. At least two independent transformants were analysed at least twice.

### Statistical methods

For each experimental study, at least two independent replicates were investigated (the number of replicates is indicated in the respective figures), and the average values displayed in a graph. The standard errors are indicated as error bars in the respective figures. A two-tailed t-test was carried out to determine whether the means of two groups were significantly different. If two groups had a p-value of under 0.05, they were considered as having statistically different means (with a confidence value of at least 95%).

## Results

### Identification of amino acids in the RWDBD relevant for causing 3AT sensitivity

We have shown previously that in Gcn1, Arg-2259 is critical for Gcn2 binding [5]. To predict additional amino acids relevant for Gcn2 binding, we interrogated our computationally modelled structure of the RWDBD [20], to identify surface exposed amino acids that are in close proximity to Arg-2259. Since Arg-2259 is a charged amino acid, and is located within a patch of similarly charged amino acids [20], we reasoned that these amino acids may also be involved in Gcn2 binding. These amino acids, as well as other amino acids as described below, were chosen for further investigation.

In order to validate whether these selected amino acids may be required for Gcn2 binding, we took advantage of the fact that Gcn1-Gcn2 interaction is absolutely required for Gcn2 activation in response to amino acid starvation [5]. Here we employed an assay we had established previously that allows for the easy detection of amino acids relevant for Gcn1-Gcn2 interaction and Gcn2 activation [5, 23]. In this assay, plasmid-borne GST-tagged RWDBD was overexpressed in yeast from a galactose inducible promotor. We have established previously, that RWDBD competes with endogenous Gcn1 for Gcn2 binding, impairing Gcn1-Gcn2 interaction, and thereby hampers Gcn2 activation, i.e. RWDBD overexpression elicits a dominant negative effect. This dominant negative effect can be easily scored by growing cells on medium containing 3-amino-2,4-triazole (3AT). 3AT inhibits an enzyme in the His biosynthetic pathway, thereby eliciting His starvation [28]. Only cells able to activate Gcn2 can overcome starvation and grow, while strains unable to activate Gcn2 cannot grow.

**Fig 1:**
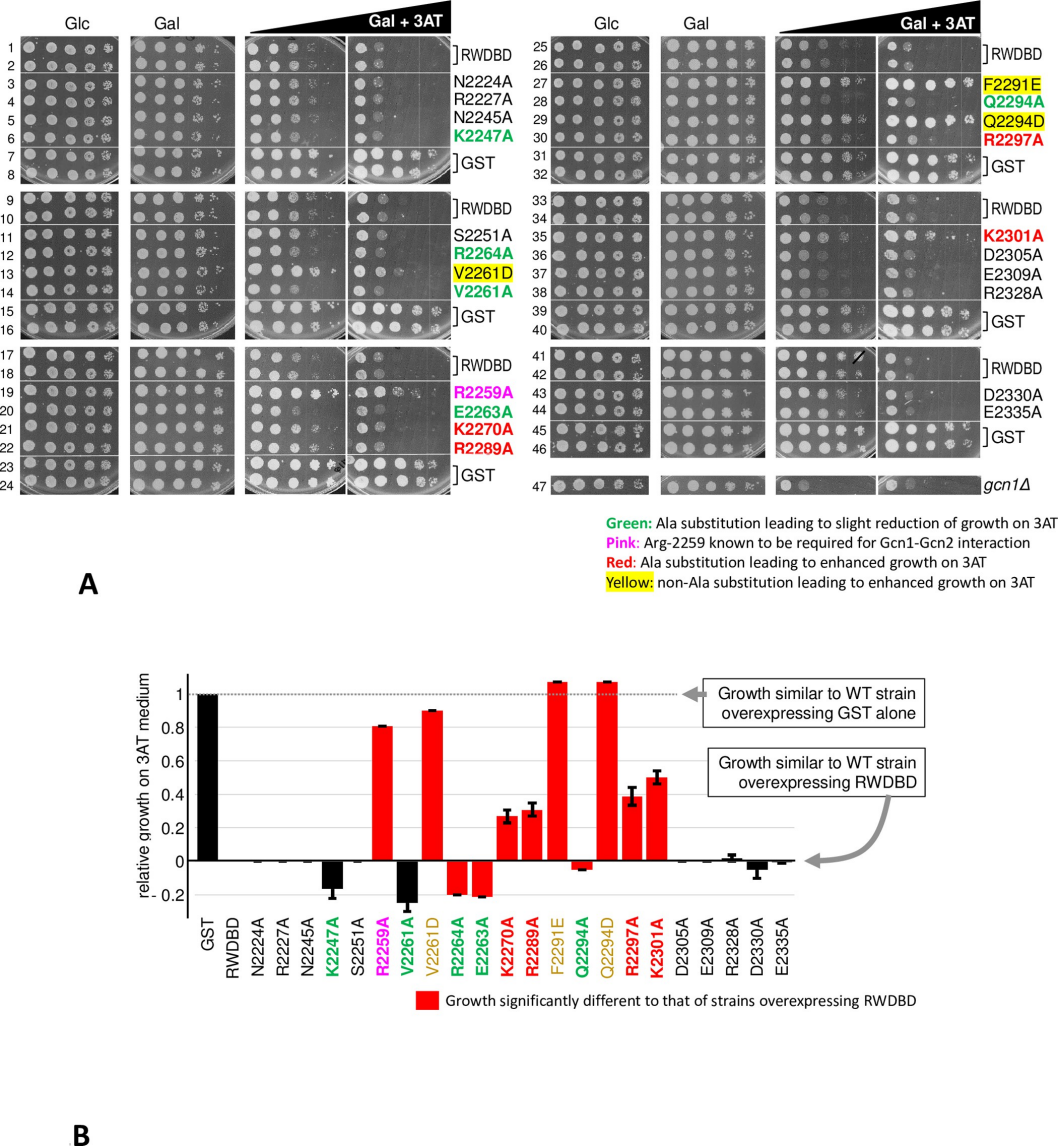
Screening for single amino acid substitutions that modulate the dominant negative effect elicited by the overexpressed RWDBD. **(A)** Wild-type yeast strain H1511 was transformed with plasmids expressing GST alone or GST-tagged RWDBD from a galactose inducible promotor. The RWDBD was either un-mutated (RWDBD), or contained single amino acid substitutions as indicated. As control, the isogenic *gcn1Δ* strain H2556 was transformed with GST alone. Transformants were grown to exponential phase, subjected to 10-fold serial dilutions, and aliquots were transferred to solid medium containing glucose (Glc) or galactose (Gal) as carbon source, as well as 3-amino-1,2,4-triazole (3AT), as indicated. 3AT concentrations were 30 and 120 mM (left and right panels underneath the black triangle labelled Gal+3AT). The assays were performed at least 4 times, and a sample result is shown. The same strains were grown on Glucose and Glucose + 3AT medium (not shown). Under these conditions, where the RWDBD variants are not overexpressed, the 3AT resistance of H1511 was not altered, suggesting that the effects seen on Gal+3AT are truly due to the overexpression of the RWDBD variants. **(B)** The growth in (A), from four independent experiments, was quantified as outlined in detail in the materials and methods section. Briefly, for each growth assay (rows 1–8, 9–16, 17–24, 25–32, 33–40, 41–46), the growth of each strain on each Gal+3AT plate was determined relative to that on Gal plates. Then, since the difference in growth between strains overexpressing GST alone and RWDBD were not the same between the growth assays, we next set the growth rate of the strain overexpressing RWDBD to zero by subtracting its growth value from that of all the other strains. After that, the relative growth rate was determined, relative to the strain overexpressing GST alone, and plotted on a bar graph. Error bars indicate the standard errors, and growth rates significantly different to that of the strain overexpressing un-mutated RWDBD are indicated in red (Student two-tailed t-test, p < 0.05).

**Fig 2:**
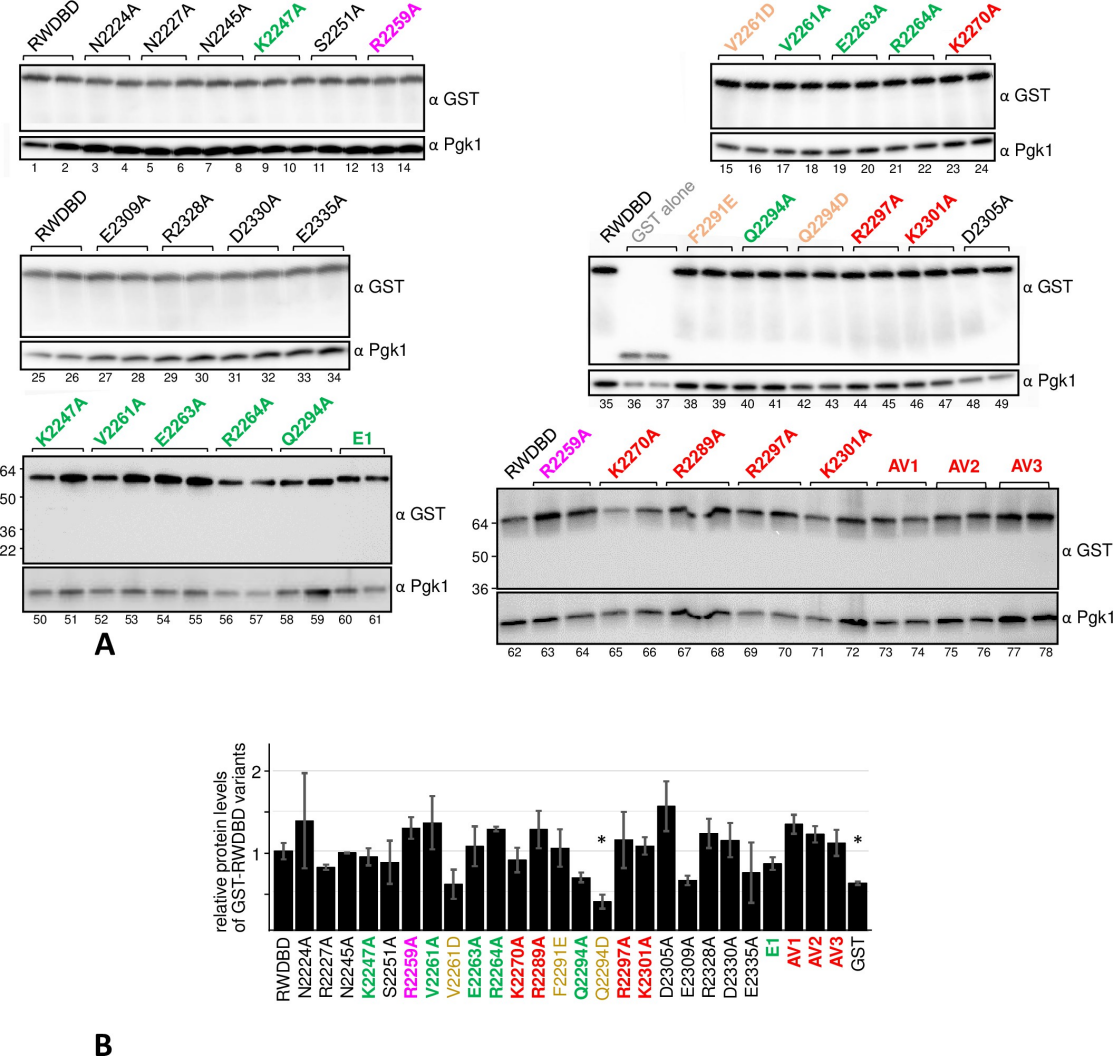
Determining the protein levels of the RWDBD variants. **(A)** Strains as indicated were grown to exponential phase, exposed to formaldehyde before harvesting, and subjected to western blotting using antibodies against the GST-tag present on all RWDBD variants, and against Pgk1 as control for equal loading. Four independent experiments were conducted and representative images are shown. **(B)** Signals in (A), obtained from four independent experiments, were quantified using Image J, and for each sample the GST signal was divided by the Pgk1 signal. For each strain, this ratio was divided by the ratio of the strain expressing wild-type RWDBD. These results were plotted in a bar graph, and the standard errors are shown as error bars. Values significantly different to that of RWDBD are indicated with an asterisk (Student two-tailed t-test, p < 0.05).

Wild-type yeast H1511 was transformed with plasmids each expressing an RWDBD variant containing one single amino acid substitution. In addition to these RWDBD variants, as control H1511 was separately transformed with plasmids expressing GST alone or un-mutated RWDBD. The resulting transformants were subjected to semi-quantitative growth assays, as described in the materials and methods section. In this growth assay, solid medium was used that contained glucose as carbon source, or galactose to induce the overexpression of the plasmid borne RWDBD variants or GST alone. In addition, galactose containing medium was used that contained 3AT at different concentrations (Fig 1A). In parallel, to ensure that the observed phenotypes were not due to differences in expression levels of the RWDBD variants, we scored their protein levels. For this, strains were grown to exponential phase, harvested, and the cell extracts subjected to denaturing SDS-polyacrylamide gel electrophoresis (SDS-PAGE), and western blotting using antibodies against the GST tag located at the N-terminus of all RWDBD constructs, and against the housekeeping gene Pgk1 as a control for equal loading (Fig 2).

On medium containing galactose but not 3AT, all strains grew equally well as found on medium containing glucose as carbon source, suggesting that overexpression of RWDBD did not impact the cell growth in general. On galactose medium containing 3AT, we found that some mutations in the RWDBD had varying effects on cell growth. In order to more easily compare the differences in growth, we quantified the amount of growth in the presence of 3AT as outlined in the materials and methods section (Fig 1B). As expected, overexpression of the wild-type RWDBD led to impaired growth on 3AT medium, i.e. it elicited a 3AT sensitivity (3AT^s^) phenotype, as compared to strains overexpressing GST alone (Fig 1A, e.g. rows 1,2 vs 7,8).

We found that amino acid substitutions K2270A, R2289A, R2297A, and K2301A in the RWDBD, respectively, each led to improved growth on 3AT medium, i.e. they reverted the 3AT^s^ phenotype, at least to some extent (Fig 1A, rows 21&22 vs 17&18, 30 vs 25&26, 35 vs 33&34, Fig 1B), as found previously for the R2259A substitution (Fig 1A, row 19 vs 17&18, [5]). The growth rates appeared to be significantly different from that elicited by wild-type RWDBD (Fig 1B), suggesting that the mutated amino acids reverted the dominant negative effect. The amino acid substitutions R2227A, K2247A, R2264A, and R2328A, respectively, did not revert the growth defect associated with overexpression of the RWDBD (Fig 1A, rows 4&6 vs 1&2, 12 vs 9&10, 38 vs 33&34), despite of being overexpressed as well as wild-type RWDBD (Fig 2). This suggested that these amino acids are not relevant for the RWDBD to elicit a dominant negative phenotype.

As a control, we have conducted Ala substitutions of a selection of amino acids that were oppositely charged to Arg-2259, which were Glu-2263, Asp-2305, Glu-2309, Asp-2330, and Glu-2335. We found that none of these reverted the dominant negative property of the RWDBD (Fig 1, row 20 vs 17&18, 36&37 vs 33&34, 43&44 vs 41&42, Fig 1B). Similarly, Ala substitutions of a selection of polar amino acids, Asn-2224, Asn-2245, Ser-2251, and Gln-2294 (Fig 1, rows 3&5 vs 1&2, 11 vs 9&10, 28 vs 25&26, Fig 1B), did not revert the dominant negative property of the RWDBD.

In contrast to the Ala substitutions, the V2261D and Q2294D substitutions each reverted the dominant negative phenotype associated with overexpression of the RWDBD, (Fig 1A, row 14 vs 13, row 28 vs 29, Fig 1B), which may suggest that the introduced negative charge interferes with the RWDBD-Gcn2 interaction. Though it shall be noted that RWDBD-Q2294D was expressed significantly lower than the un-mutated RWDBD (Fig 2B), thus we cannot exclude the possibility that the low expression level of this variant is the reason for its inability to cause a dominant negative phenotype.

Phe-2291 is buried inside the RWDBD, and appears to be involved in maintaining the RWDBD three-dimensional structure [20]. If that is the case, then substitution of Phe with a charged amino acid, Glu, should affect the proper folding of the RWDBD, rendering it unable to disrupt Gcn1-Gcn2 interaction. In fact, in agreement with this idea, RWDBD-F2291E was unable to elicit a dominant negative effect (Fig 1A, rows 27 vs 25&26, Fig 1B).

### Four amino acids in the GST-RWDBD appear to be important for Gcn2 inhibition

**Fig 3:**
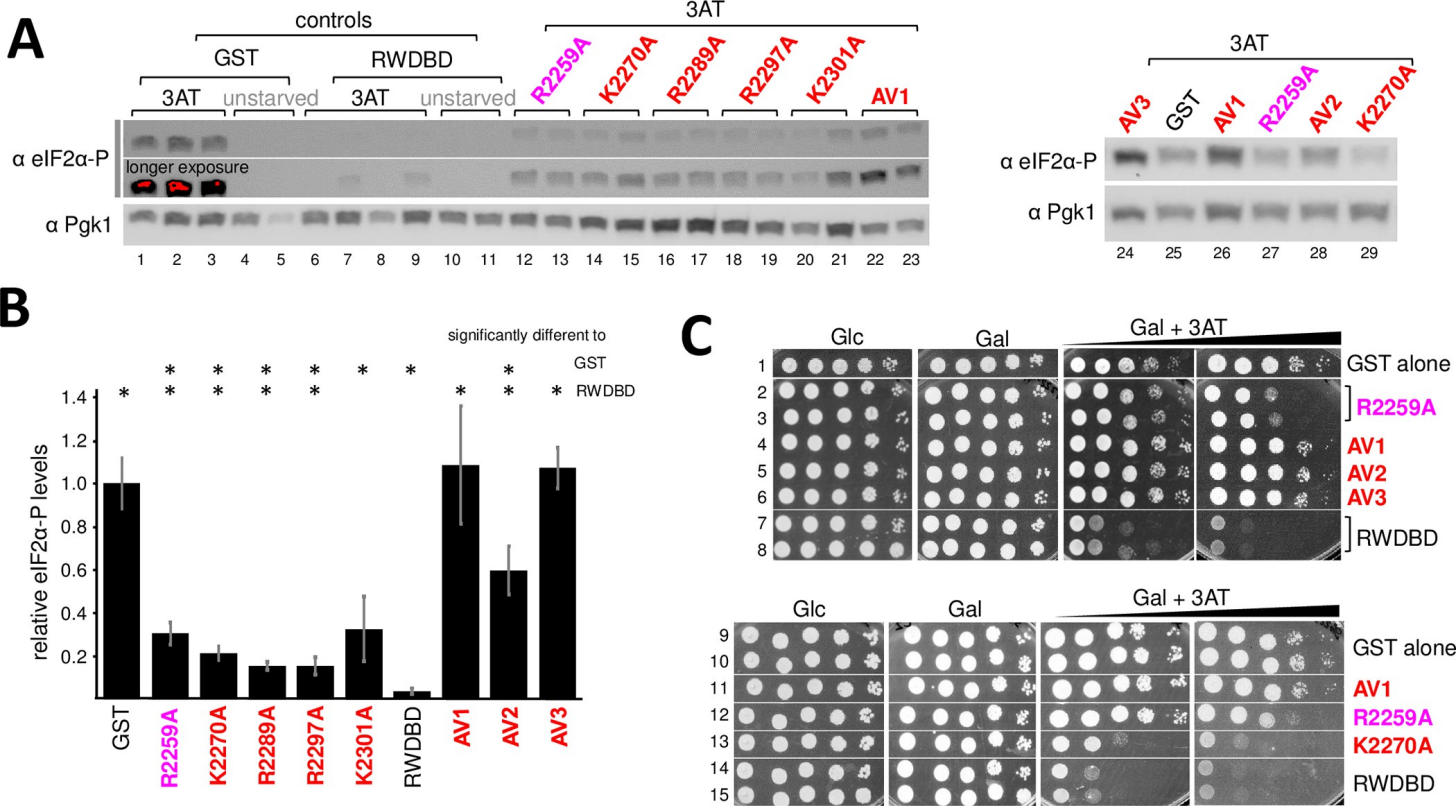
Analysis of multiple amino acid substitutions targeting residues that each impaired the dominant negative effect of the RWDBD. **(A)** Strains overexpressing RWDBD variants or GST alone as indicated, were grown to exponential phase before adding 30 mM 3AT (final concentration) for 30 min. Cells were exposed to formaldehyde before harvesting, and cell extracts were subjected to SDS-PAGE and immunoblotting using antibodies specific against phosphorylated eIF2α (eIF2α-P), and against Pgk1 as control for equal loading. Representative images are shown from at least 4 independent experiments. **(B)** The relative level of eIF2α-P in (A), from four independent experiments, was determined as done in Fig 2, just that the eIF2α-P signal was divided by the Pgk1 signal. Error bars indicate standard errors. Values significantly different to that of GST or RWDBD are indicated with asterisks (student two-tailed t-test, p < 0.05). **(C)** The strains in (A) were subjected to semi-quantitative growth assays as done in Fig 1. For medium containing Galactose and 3AT (Gal+3AT), plates containing 30 mM and 120 mM 3AT are shown (Left and right panel underneath the black triangle labelled Gal+3AT). Images are representative results for at least four independent experiments.

The amino acid substitutions K2270A, R2289A, R2297A, and K2301A, respectively, reversed the dominant negative effect of overexpressed RWDBD, at least to some extent, visible by improved growth on 3AT medium as compared to a strain overexpressing wild-type RWDBD (Fig 1A and 1B). In order to validate that this was truly due to the reversion of RWDBD-mediated Gcn2 inhibition, we scored for the levels of phosphorylated eIF2α, the substrate of Gcn2. For this, cells were grown to exponential phase in galactose containing medium, and then 30 mM 3AT (final concentration) was added for 30 min. Cells were then treated with formaldehyde before harvesting, and the generated cell extracts were subjected to SDS-PAGE and immunoblotting using antibodies specific against phosphorylated eIF2α (eIF2α), and against Pgk1 as control for equal loading. Under starvation conditions, as published previously [5], cells expressing GST alone showed a robust increase in the level of eIF2α-P (Fig 3A, lanes 1–3 vs 4&5), while overexpression of the RWDBD led to a weaker increase in eIF2α-P levels (Fig 3A, lanes 1–3 vs 6–9). However, K2270A, R2289A, R2297A, and K2301A substitutions, respectively, led to eIF2α-P levels that were higher than that in strains overexpressing un-mutated RWDBD (Fig 3A lanes 6–9 vs 14–21, Fig 3B), suggesting that Gcn2 activation was restored, at least to some extent. This phenomenon was similar to that observed for the R2259A substitution in the RWDBD (Fig 3A lanes 6–9 vs 12&13, Fig 3B) [5]. This in agreement with the idea that Lys-2270, Arg-2289, Arg-2297, and Lys-2301 in the RWDBD are each necessary to elicit a dominant negative phenotype.

### At least two amino acids in the RWDBD co-operate in eliciting the dominant negative effect

The amino acid substitutions R2259A, K2270A, R2289A, R2297A, and K2301A, each reverted the dominant negative 3AT^s^ phenotype elicited by the RWDBD, but only in part, raising the possibility that the respective amino acids co-operate in Gcn2 binding. If that is true, then combining all substitutions should lead to a stronger reversion of the phenotype. To test this, we substituted all five amino acids in the RWDBD, and the resulting RWDBD variant was dubbed RWDBD-AV1. In semiquantitative growth assays we found that the dominant negative effect of the RWDBD was reverted more by the AV1 substitutions than by R2259A (Fig 3C, row 4 vs 2&3, 12 vs 11), and this was not due to the reduced expression of RWDBD-AV1 (Fig 2). It appeared that strains overexpressing RWDBD-AV1 grew almost as well as strains overexpressing GST alone (Fig 3C, row 4 vs 1, row 11 vs 9&10). Under amino acid starvation, strains overexpressing RWDBD-AV1 seemed to have higher eIF2α-P levels than strains overexpressing RWDBD-R2259A (Fig 3A, lanes 22&23 vs 12&13, lanes 26 vs 27, Fig 3B), in fact the eIF2α-P levels appeared to be similar to that of strains overexpressing GST alone. This would support the idea that the substituted amino acids co-operate in mediating Gcn2 binding.

Of those five substituted amino acids, Lys-2270 appears to be buried inside the RWDBD, suggesting that it is relevant for maintaining proper RWDBD folding [20]. The Arg-2289 side chain is oriented towards a different surface side of Gcn1 as compared to Arg-2259, Arg-2297, and Lys-2301 [20]. Therefore, we re-introduced the amino acids Lys-2270 and Arg-2289, respectively, into RWDBD-AV1. The effect of the resulting constructs, dubbed RWDBD-AV2 and RWDBD-AV3, respectively, were tested in semiquantitative growth assays for their ability to elicit a 3AT^s^ phenotype, and tested via immunoblotting for their effect on eIF2α-P levels. We found that strains overexpressing RWDBD-AV2 and RWDBD-AV3, respectively, grew as well as strains overexpressing RWDBD-AV1 (Fig 3C, row 4 vs 5&6), and this was not due to reduced protein levels of the RWDBD variants (Fig 2). When quantifying the eIF2α-P levels from several westerns, it appeared that overexpression of RWDBD-AV3 (no R2289A substitution) elicited eIF2α-P levels comparable to that of strains overexpressing GST alone or overexpressing RWDBD-AV1 (Fig 3B), suggesting that RWDBD-AV3 has completely lost its ability to impair Gcn2 activation as found for RWDBD-AV1. Strains overexpressing RWDBD-AV2 (no K2270A substitution) showed higher eIF2α-P levels than strains overexpressing RWDBD-R2259A (Fig 3A, lane 27 vs 28, Fig 3B). However, the eIF2α-P levels were not fully reverted back to that of strains overexpressing RWDBD-AV1 or GST alone (Fig 3B), suggesting that RWDBD-AV2 is still able to inhibit Gcn2 to some extent. These findings will be addressed further in the discussion section.

### Full length Gcn1-AV2 is unable to rescue the 3AT^s^ phenotype of a *gcn1Δ* strain

**Fig 4:**
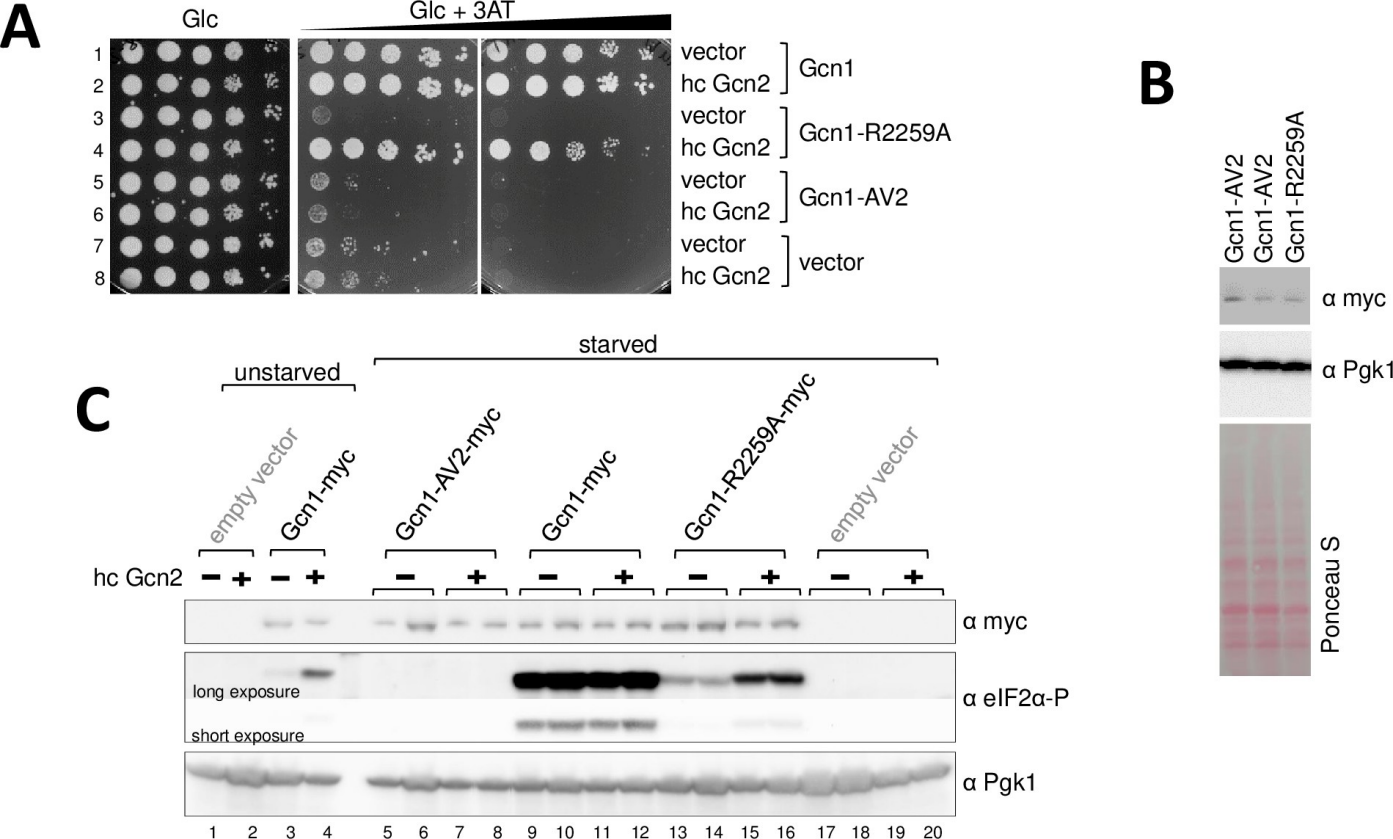
Full length Gcn1-AV2 is unable to confer growth on 3AT^s^ medium. **(A)** The *gcn1Δ* strain H2556 was transformed with a low copy plasmid expressing myc tagged full-length Gcn1, Gcn1-R2259, Gcn1-AV2, or no protein, and a high copy plasmid expressing Gcn2 (hc Gcn2) or no protein. The resulting transformants were subjected to semi-quantitative growth assays as done in Fig 1, using plates containing glucose as carbon source (Glc) or glucose and 3AT (Glc+3AT, 60 and 150 mM 3AT). **(B)** The Gcn1-AV2 is expressed. The *gcn1Δ* strain H2556 expressing either myc-tagged Gcn1-AV2 or Gcn1-R2259A, was grown in liquid medium to exponential phase, harvested, and the cell extract subjected to western blotting as done in Fig 2B, just that antibodies against the myc tag and against Pgk1 were used. The PonceauS stained membrane is shown as an additional control for equal loading. Images are representative results for two independent experiments. **(C)** The AV2 mutations impair eIF2α-P levels more so than the R2259A substitution. The cells from (A) were subjected to starvation via the addition of 3AT, and the cell extracts were subjected to western blotting as done in Fig 3A, using antibodies against eIF2α-P, Pgk1, and the myc epitope present at the C-terminus of the Gcn1 proteins.

Having established that the AV2 mutations severely reduced the ability of the RWDBD to inhibit Gcn2, more so than the single R2259A substitution, we next wanted to investigate the effect of the AV2 mutations in the context of full-length Gcn1. We have shown previously, that Gcn1-R2259A is unable to rescue the 3AT^s^ phenotype of a *gcn1Δ* strain, but is able to do so in part when Gcn2 is overexpressed in the cell [5]. Overexpression of Gcn2 likely has driven–by mass action–the interaction between Gcn2 and Gcn1-R2259A, thereby rescuing in part Gcn2 activation. Using this assay, we wanted to test whether Gcn1-AV2, containing amino acid substitutions in addition to the R2259A substitution, is able to rescue the 3AT^s^ phenotype of a *gcn1Δ* strain. For this, we transformed the *gcn1Δ* strain H2556 with a high copy plasmid expressing Gcn2 from its native promotor or the empty vector, and a low copy plasmid expression from its native promotor myc-tagged Gcn1, Gcn1-R2259A, Gcn1-AV2, or vector alone. The resulting transformants were subjected to semi-quantitative growth assays as done above, using plates containing 3AT or not. As expected, in contrast to cells containing wild-type Gcn1, cells lacking Gcn1 were hardly able to grow on 3AT, even when Gcn2 was overexpressed (Fig 4, Rows 1&2 vs 7&8). As published previously [5], cells harbouring Gcn1-R2259A were only able to grow on 3AT medium when Gcn2 was overexpressed (Fig 4, Rows 3 vs 4). In contrast to this, we here found that cells harbouring Gcn1-AV2 were unable to grow on 3AT medium, and this could not be rescued by Gcn2 overexpression (Fig 4, rows 5 vs 6). Western blotting demonstrated that lack of complementing the 3AT^s^ phenotype of a *gcn1Δ* strain was not due to lack of Gcn1-AV2 expression (Fig 4B).

To test whether the differences in growth rates on 3AT medium (Fig 4A) were truly due to the differences in the level of Gcn2 activation, we next scored for the phosphorylation levels of the Gcn2 substrate, eIF2α. For this, the above transformants (Fig 4A) were grown to exponential phase, exposed to 3AT (to induce starvation) before harvesting, and cell extracts subjected to western blotting as done above. Cells containing Gcn1-myc showed a robust increase in eIF2α-P levels under starvation (Fig 4C, lane 3 vs 9&10). Overexpression of Gcn2 increased the basal eIF2α-P levels under unstarved conditions (Fig 4C, lane 3 vs 4), but did not further increase the eIF2α-P levels under starved conditions (Fig 4C, lanes 9&10 vs 11&12). On the other hand, under starvation conditions, cells expressing Gcn1-R2259A showed lower eIF2α-P levels than cells expressing wild-type Gcn1 (Fig 4C, lanes 13&14 vs 9&10). Overexpression of Gcn2 lead to a further increase in eIF2α-P levels in the Gcn1-R2259A strains (Fig 4C, lanes 13&14 vs 15&16), but the levels were still lower than that of cells expressing wild-type Gcn1 (Fig 4C, lanes 15&16 vs 11&12). This is in agreement with our previous findings that the R2259A substitution impairs Gcn2 activation due to reduced Gcn1-Gcn2 interaction [5], and that overexpression of Gcn2 rescued the 3AT^s^ phenotype of the Gcn1-R2259A strain in part (Fig 4A, row 3 vs 4 vs 1&2) [5]. Cells expressing Gcn1-AV2 didn’t show detectable levels of eIF2α-P under starvation conditions, even when Gcn2 was overexpressed in the cell (Fig 4C, lanes 5&6 vs 7&8), in agreement with the idea that the AV2 mutations have severely impaired Gcn1-Gcn2 interaction.

### Amino acid substitutions that increase the dominant negative effect elicited by the RWDBD

**Fig 5:**
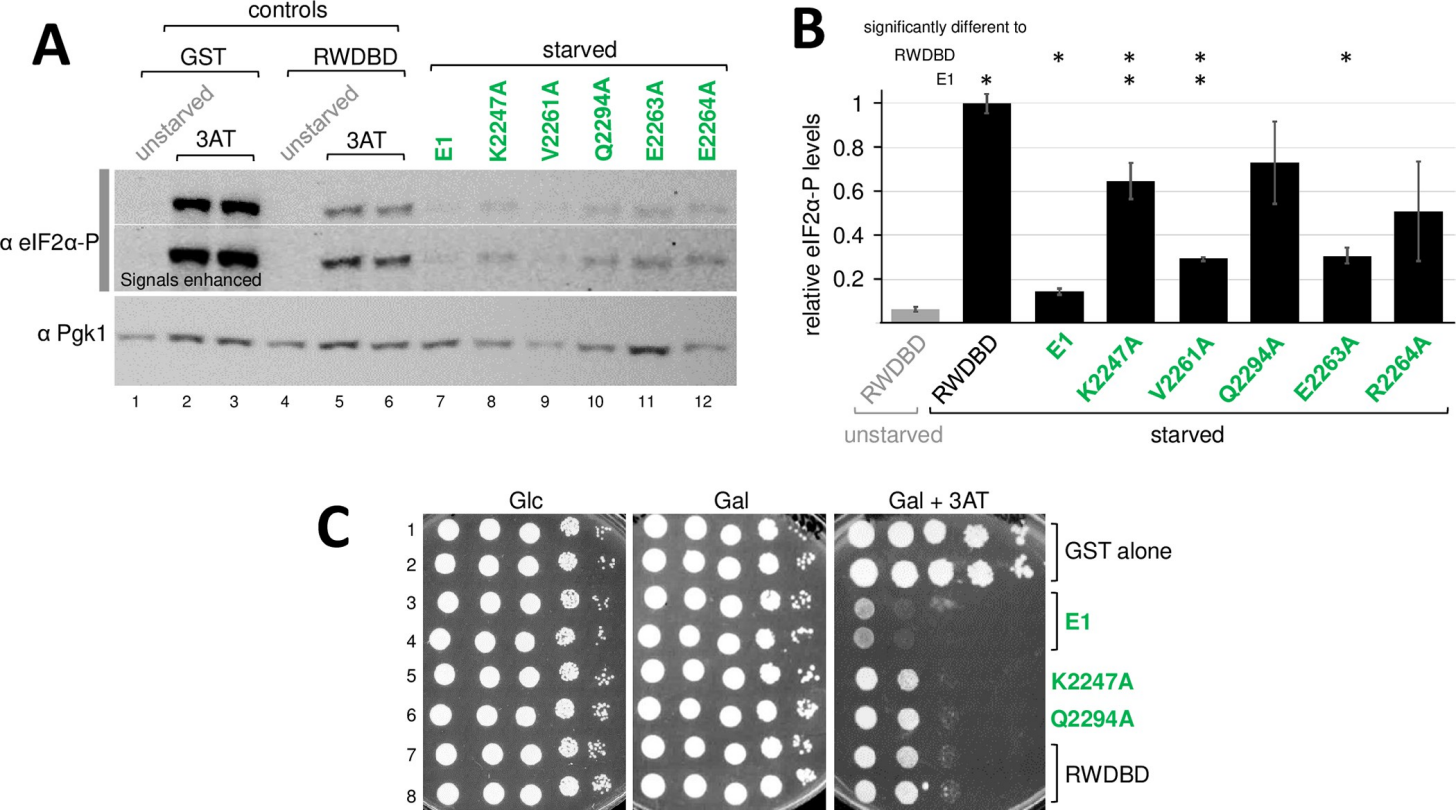
Analysis of multiple amino acid substitutions targeting residues that each enhanced the dominant negative effect of the RWDBD. **(A)** Strains overexpressing RWDBD variants or GST alone as indicated were subjected to immunoblotting assays to determine eIF2α-P levels as done in Fig 3. At least five independent experiments were conducted, and a representative result is shown. **(B)** The relative level of eIF2α-P level in (A) was determined as done in Fig 3, and standard errors are shown as error bars. Values significantly different to that of the RWDBD are indicated with an asterisk (Student two-tailed t-test, p < 0.05). **(C)** The strains in (A) were subjected to semi-quantitative growth assays as done in Fig 1A. At least three independent experiments were conducted, and a representative result is shown.

We serendipitously found that five amino acid substitutions, K2247A, V2261A, E2263A, R2264A, and Q2294A, each seemed to have increased the 3AT^s^ phenotype as compared to the strain overexpressing un-mutated RWDBD (Fig 1A, rows 6, 12, 14, 20, 28). For at least three of these substitutions, these differences were significant (Fig 1B). This seemed to suggest that these RWDBD variants are more potent in binding and inhibiting Gcn2. Supporting this idea, when scoring for eIF2α-P levels, we found that the RWDBD each containing one of the above single amino acid substitutions led to a further reduction in eIF2α-P levels as compared to wild-type RWDBD (Fig 5A, lane 5&6 vs 8–12, Fig 5B).

Next, we tested whether an RWDBD containing all five amino acid substitutions, dubbed RWDBD-E1, would further enhance the dominant negative effect and further decrease the eIF2α-P levels. In fact, it appeared that RWDBD-E1 overexpression led to weaker growth on 3AT containing medium than overexpression of RWDBDs with single amino acid substitutions, such as RWDBD-K2247A and RWDBD-Q2294A (Fig 5C, row 3,4 vs 5&6). This was not due to enhanced expression levels of RWDBD-E1 (Fig 2B). Furthermore, we found that the eIF2α-P levels elicited by RWDBD-E1 appeared to be even lower than those elicited by the RWDBD variants containing single amino acid substitutions (Fig 5A, lane 7 vs 8–12, Fig 5B). Together, this suggests that these amino acid substitutions render the RWDBD more potent in preventing Gcn2 activation.

## Discussion

Past studies have suggested that in Gcn1 more amino acids than just Arg-2259 are required for Gcn2 binding [5]. This study aimed to identify these additional amino acids, using a genetic approach. A fragment in Gcn1 called the RWDBD has been shown previously to harbour the Gcn2 binding site, and to be sufficient for disrupting Gcn1-Gcn2 interaction *in vivo* when overexpressed [5]. As a consequence, Gcn2 cannot be activated in an otherwise wild-type strain, visible by reduced growth under starvation conditions and by reduced eIF2α-P levels. This dominant negative phenotype elicited by the RWDBD is reverted by the R2259A substitution in the RWDBD. We had established that this amino acid substitution weakens the affinity of the RWDBD for Gcn2, thereby allowing endogenous Gcn1-Gcn2 interaction again to occur in the cell [5]. In agreement with the idea that amino acids in addition to Arg-2259 mediate Gcn2 binding, here we found that the RWDBD-R2259A still caused a dominant negative phenotype, albeit to a small extent, and that it still hampered to some extent the increase in eIF2α-P levels. Thus, RWDBD-R2259A had not completely lost its affinity to Gcn2.

A past study had conducted a mutagenesis screen to find amino acids in Gcn1 that mediate Gcn1-Gcn2 interaction [29]. This work found that the Gcn1 F2291L substitution impaired Gcn1-Gcn2 interaction as well as led to reduced eIF2α-P levels. Phe-2291 appears to be buried in the protein [20], suggesting that its substitution destabilises the RWDBD structure and thereby prevents Gcn1-Gcn2 interaction. Hence, Phe-2291 likely is not a Gcn2 binding site *per se*. Two additional amino acid substitutions were found in this screen that abolished Gcn1-Gcn2 interaction, S2304P and L2353P. With being a secondary amide, Pro can affect the protein’s secondary structure. Therefore, it is possible that these substituted amino acids are not direct Gcn2 contact points, but instead the Pro substitution has compromised the RWDBD structure, thereby indirectly impairing Gcn1-Gcn2 interaction. The mutagenesis screen used a sophisticated approach to remove any mutations that lead to truncated proteins, however, this screening procedure did not allow the removal of amino acid substitutions impacting on the RWDBD folding. The abundant occurrence of mutations impacting the three-dimensional structure of the RWDBD may have been the reason why no amino acids were found that mediated Gcn2 binding *per se*, such as Arg-2259 [5].

For that reason, we chose an alternative approach for detecting candidate amino acids in the RWDBD that are required for Gcn2 binding. Using our modelled RWDBD structure [20], we first predicted amino acids that may contact Gcn2 using three criteria. Firstly, we reasoned that additional contact points are likely in close proximity to Gcn1 Arg-2259, a verified contact point for Gcn2 [5]. Secondly, the amino acids mediating the interaction are positively charged. This reasoning came from studies on Yih1. Yih1 also contains an RWD domain as found for Gcn2 [19], and we have shown that Yih1 competes with Gcn2 for Gcn1 binding (5), suggesting that both utilise the same binding properties in Gcn1 [5]. In Yih1 two amino acids have been identified that are required for Gcn1 binding, Asp-102 and Glu-106 [12]. These amino acids are oppositely charged to Arg-2259, suggesting that Gcn1-Gcn2 interaction is an ionic interaction, and that in Gcn1 amino acids contacting Gcn2 are positively charged. Thirdly, amino acids contacting Gcn2 should be surface exposed.

We substituted a series of positively charged amino acids by Ala, but also as controls substituted a selection of negatively charged, polar and hydrophobic residues. We found three amino acid substitutions that each reverted the dominant negative phenotype of RWDBD. These also allowed again increased levels of eIF2α-P, suggesting that Gcn2 function was restored, in agreement with the idea that these RWDBD variants were less able to bind to Gcn2 to disrupt Gcn1-Gcn2 interaction. In agreement with our prediction, the substituted amino acids were all positively charged, Arg-2289, Arg-2297, and Lys-2301. This supports our idea that the ionic Gcn1-Gcn2 interaction is facilitated by positively charged residues in Gcn1 and negatively charged counterparts in Gcn2. These three amino acids appear to be specific contact points, because other positively charged amino acids, Arg-2227, Lys-2247, Arg-2264, and Arg-2328, did not lead to a reversion of the dominant negative property of the RWDBD. One could argue that the more relevant a Gcn1 amino acid is for mediating Gcn2 binding, the more should its substitution revert the dominant negative phenotype of the RWDBD. In this scenario Arg-2259 would be most relevant, followed by Lys-2301, followed by the other two amino acids (Fig 1B).

**Fig 6:**
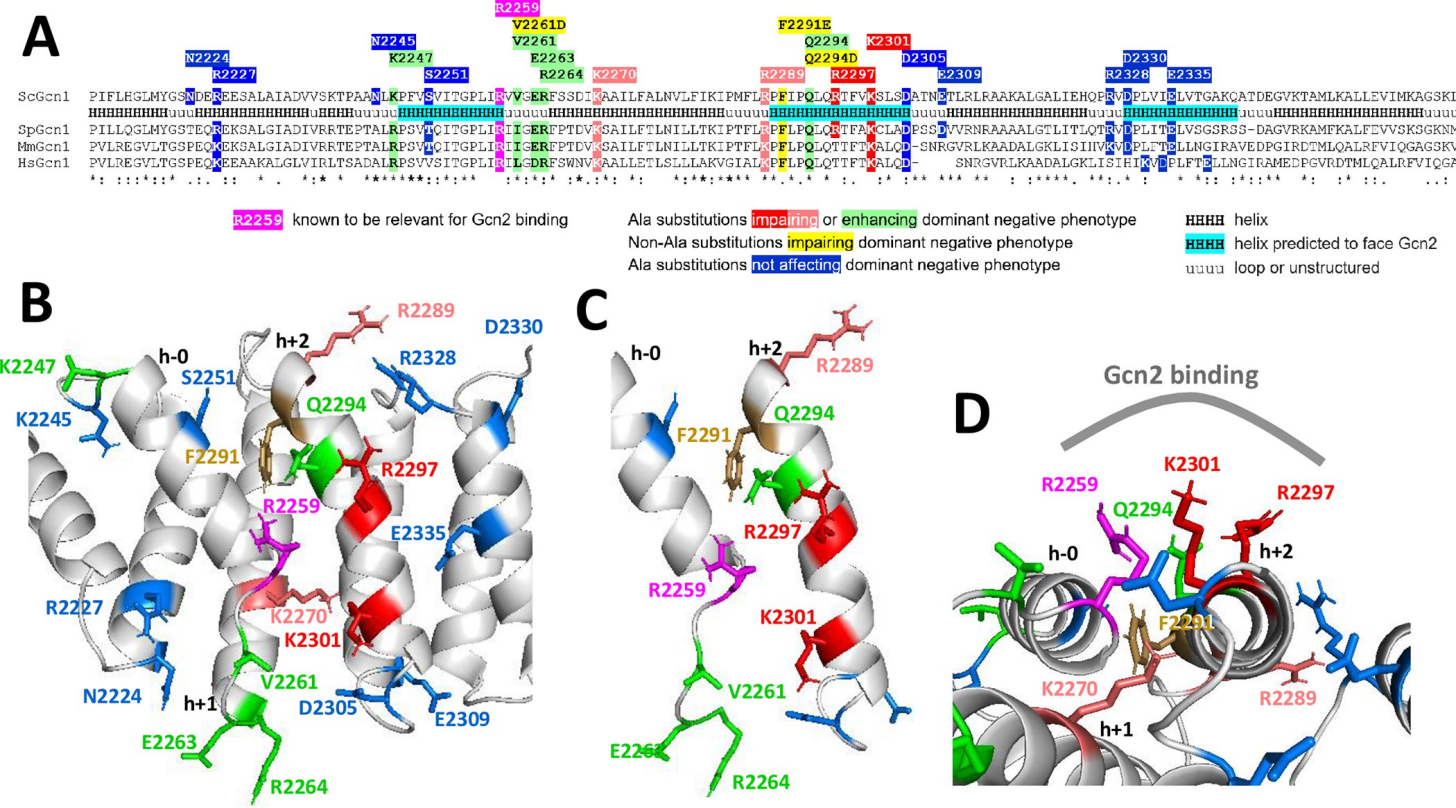
Location of substituted amino acids in Gcn1. **(A)** Multiple sequence alignment of Gcn1. Gcn1 proteins from *Saccharomyces cerevisiae*, *Schizosaccharomyces pombe*, *Mus musculus*, *and Homo sapiens* (accession numbers P33892, Q10105, AAI50736.1, NP_006827.1) were subjected to a multiple sequence alignment using Clustal omega [31]. A portion of the alignment covering the area surrounding yeast Gcn1 Arg-2250 is shown. Beneath the *S*. *cerevisiae* Gcn1 sequence its secondary structure is shown as modelled by [20]. Helixes predicted to face Gcn2 are highlighted in cyan. Amino acid substitutions investigated in this work are highlighted and the key for the colours is provided in the image. **(B)** Shown is a portion of the modelled structure of the *S*. *cerevisiae* RWDBD [20], and amino acids investigated are highlighted as in (A). **(C)** Same image as in (B), just that only the helix adjacent to Arg-2259 (dubbed h-0) and the neighbouring helix (h+2) harbouring Arg-2289, Arg-2297 and Lys-2301 are shown. **(D)** Side view of the helices h-0 and h+2, and the potential Gcn2 interaction side. Part of helix h+1 can be seen which connects helix h-0 with helix h+2.

The K2270A substitution also reverted the dominant negative phenotype associated with RWDBD overexpression, as well as allowed again increased eIF2α-P levels. Its side-chain appeared to contact amino acid side-chains within Gcn1 (Fig 6B and 6D), suggesting that it is critical for maintaining the correct folding of the RWDBD. Hence, it is less likely that it is a Gcn2 contact point *per se*.

We subjected the RWDBD to multiple amino acid substitutions. The RWDBD-AV2 (R2259A, R2289A, R2297A, and K2301A) severely lost its ability to cause a dominant negative phenotype, as determined in semi-quantitative growth assays. However, while strains overexpressing RWDBD-AV2 showed increased eIF2α-P levels as compared to the strains overexpressing un-mutated RWDBD, the eIF2α-P levels were not as high as that of strains overexpressing GST alone. This suggested that RWDBD-AV2 still contains a Gcn2 contact point. Interestingly though, the eIF2α-P levels in strains overexpressing RWDBD-AV2 was higher than that of strains overexpressing the RWDBD variants containing the respective single amino acid substitutions, suggesting that the mutated amino acids co-operate in contacting Gcn2. Full length Gcn1 containing the AV2 mutation was unable to complement the 3AT^s^ of a *gcn1Δ* strain, nor was it able to promote eIF2α phosphorylation. and this was still the case when Gcn2 was overexpressed in the cell, in contrast to Gcn1-R2259A [5]. This supported the idea that the amino acid substitutions in Gcn1-AV2 have further reduced the affinity to Gcn2, as compared to Gcn1-R2259A, such that even Gcn2-overexpression was unable to drive Gcn2-Gcn1-AV2 interaction via mass action. We considered to perform co-immunoprecipitation assays to validate that the AV2 mutation (which contains the R2259A substitution) affects Gcn2-Gcn1 interaction more so than the single R2259A substitution. However, such an assay was unfeasible given that Gcn1-R2259A is unable to co-immunoprecipitate Gcn2 at levels detectable in westerns [5].

RWDBD-AV1 differs from RWDBD-AV2 by harbouring in addition the K2270A substitution. As eluted earlier, Lys-2270 is likely relevant for maintaining the correct RWDBD structure, thereby indirectly abolishing RWDBD-Gcn2 interaction. The additional K2270A substitution led to eIF2α-P levels that were similar to that of strains overexpressing GST alone, suggesting that RWDBD-Gcn2 interaction was fully impaired. The same phenomenon was found for RWDBD-AV3 that also contained the K2270A substitution. Curiously, RWDBD-AV2 overexpression caused some reduction in eIF2α-P levels while RWDBD-AV1 and RWDBD-AV3 did not impair eIF2α-P levels, but yet all three RWDBD variants showed the same degree in the reversion of the dominant negative phenotype on growth assays. It is possible that eIF2α-P levels need to reach a particular threshold level that then is sufficient to elicit a full GAAC response, a phenomenon that was proposed earlier [9].

We serendipitously found amino acid substitutions in the RWDBD that elicited a stronger dominant negative phenotype, the amino acids were Lys-2247, Val-2261, Glu-2263, Arg-2264, and Gln-2294. This phenomenon was subtle but reproducible, and significant at least for three of the five substitutions. This growth phenomenon correlated with eIF2α-P levels being lower than that of a strain overexpressing wild-type RWDBD. Together, this seemed to suggest that Gcn2 was inhibited more strongly. This would imply that these RWDBD variants have a higher affinity to Gcn2, thus preventing Gcn1-Gcn2 interaction more effectively. Combining all five amino acid substitutions in one RWDBD, in RWDBD-E1, seemed to have rendered the RWDBD even more potent. RWDBD-E1 elicited a stronger dominant negative phenotype than the RWDBD variants with single amino acid substitutions. Also, the eIF2α-P level was lower than that of strains overexpressing RWDBD variants with single amino acid substitutions.

How can amino acid substitutions enhance RWDBD-Gcn2 interaction? One possibility is that the substitutions lead to some flexibility in the RWDBD, thereby allowing the Gcn2-contacting amino acids better access to Gcn2 for a stronger binding. The amino acids Val-2261, Glu-2263, and Arg-2264 are located in a loop close to Arg-2259, that connects the helix adjacent to Arg-2259 (dubbed h-0) with the downstream helix (h+1), and helix h+1 precedes the helix (h+2) that contains the other three amino acids found here to be potential Gcn2 contact points (Fig 6B and 6C). One could envision that Val-2261, Glu-2263, and Arg-2264 substitutions by one with a smaller side chain, Ala, allows some mobility of helix h-0 and Arg2259, enabling the RWDBD to adopt a conformation that allows stronger Gcn2 binding. The same may be true for Lys-2247 which is located in a loop as well, on the N-terminal side of helix h-0 (Fig 6B). It is tempting to speculate that Gcn1-Gcn2 interaction in the cell is meant to be weak, to allow regulation of Gcn1-Gcn2 interaction. A weak interaction would more easily allow the modulation of Gcn2 activity which has been shown to be critical for optimal cell function [1]. Supporting this idea, cells harbour RWD containing proteins, of which Yih1 (IMPACT in mammals) and Gir2 (DFRP2 in mammals), have already been shown to disrupt Gcn1-Gcn2 interaction to modulate Gcn2 activity [12–16, 18, 19]. We cannot exclude the possibility that the increased potency of RWDBD variants may be due to other factors than increased Gcn2 affinity. Nevertheless, this newly discovered phenomenon warrants more in-depth studies to reveal the causative mechanism.

According to our modelled structure of the RWDBD [20], the amino acids identified to potentially contact Gcn2, Arg-2289, Arg-2297, and Lys-2301, are located in a helix (dubbed h+2) adjacent to the helix (dubbed h-0) neighbouring Arg-2259, supporting the idea that those two helixes constitute an interaction site for Gcn2 (Fig 6B–6D). F2291E substitution fully abolished the dominant negative phenotype of the RWDBD and allowed full Gcn2 activation, and a similar phenomenon was found previously for the F2291L substitution [29]. Since Phe-2291 is located in helix h+2, and given that it interacts with side chains of helix h-0, this suggests that this amino acid is critical for holding these two helixes at the appropriate distance to allow Gcn2 interaction (Fig 6C and 6D). The Lys-2270 side chain in helix h+1 faces helix h+2 (Fig 6B and 6D), suggesting that it contacts residues in helix h+2. Since K2270A reduces the dominant negative property of the RWDBD, this suggests its importance in the appropriate positioning of h+2 for Gcn2 binding.

While Arg-2259, Arg-2297, and Lys-2301, are located close together on the Gcn1 surface, likely constituting a Gcn2 interaction hotspot (Fig 6B–6D), Arg-2289 is located further distant from these amino acids, with its side chain oriented toward a different direction than those of Arg-2259, Arg-2297 and Lys-2301 (Fig 6C). Thus Arg-2289 may constitute a separate Gcn2 interaction hot spot. Supporting this idea, the region in Gcn2 required for efficient Gcn1 binding encompasses not only the RWD domain, but also a region C-terminal to the RWD [30].

Since Gcn1-Gcn2 interaction is conserved from yeast to human, one would expect that the amino acids in Gcn1 mediating Gcn2 binding are conserved as well. Supporting this idea, Arg-2259, Arg-2289 and Lys-2301 are conserved (Fig 6A). Curiously, Lys-2270 is conserved as well and so is Phe-2291, which would support the idea that these are critical for maintaining the proper structure of the Gcn2-contacting area in Gcn1. However, Arg-2297 is not conserved, instead human Gcn1 has a Thr at this position. It will be interesting to investigate whether during evolution the Gcn1-Gcn2 interaction has diverged sightly between yeast and human.

Taken together, we have identified amino acids in the RWDBD that likely are Gcn2 contact points *per se*. This now warrants subsequent biochemical protein-protein interaction studies to verify that these amino acids are direct Gcn2 contact points. Given that Gcn2 has been implicated in many biological functions, and that the Gcn1-Gcn2 interaction is integral to Gcn2 activation and a point of regulation in the cell, detailed insight into this interaction will help understand how other RWD proteins modulate this Gcn1-Gcn2 interaction to adjust Gcn2 activity to the cell’s needs, and to the needs of each organ of a multicellular organism.

## Supporting information

S1 Raw images (PDF)

## Abbreviations

3AT: 3-amino-1,2,4-triazole
GAAC: General Amino Acid Control
Gcn: General control non-derepressible
slg: slow growth
RWD: a domain found in RING finger-containing proteins, WD-repeat-containing proteins, and yeast DEAD (DEXD)-like helicases
RWDBD: RWD binding domain
SDS-PAGE: SDS polyacrylamide gel electrophoresis
