# Supplementary material for: A genetic approach to identify amino acids in Gcn1 required for Gcn2 activation
Source: PLoS One. 2022 Nov 28;17(11):e0277648. doi: 10.1371/journal.pone.0277648 (PMC9704636; doi:10.1371/journal.pone.0277648)
Supplement: S1 Raw images — (PDF) [file pone.0277648.s001.pdf]

α GST
overlaid with size marker
Mirror image shown in Figure 2A

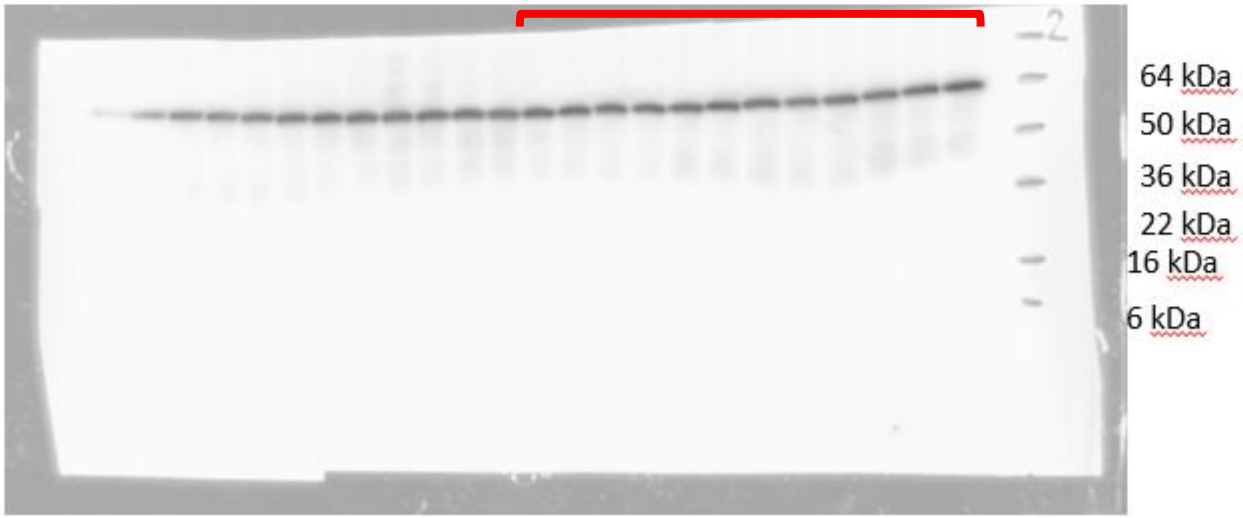

α GST
Raw image

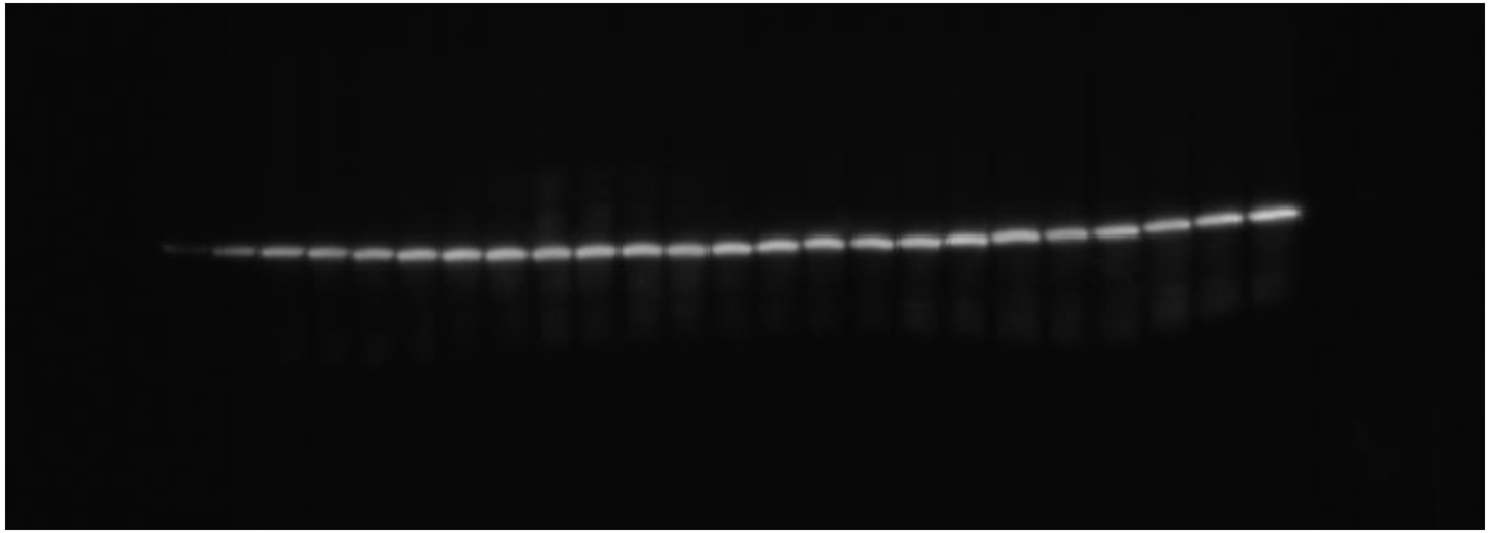

Figure 2A, top left western

α Pgk1
overlaid with size marker
Mirror image shown in Figure 2A

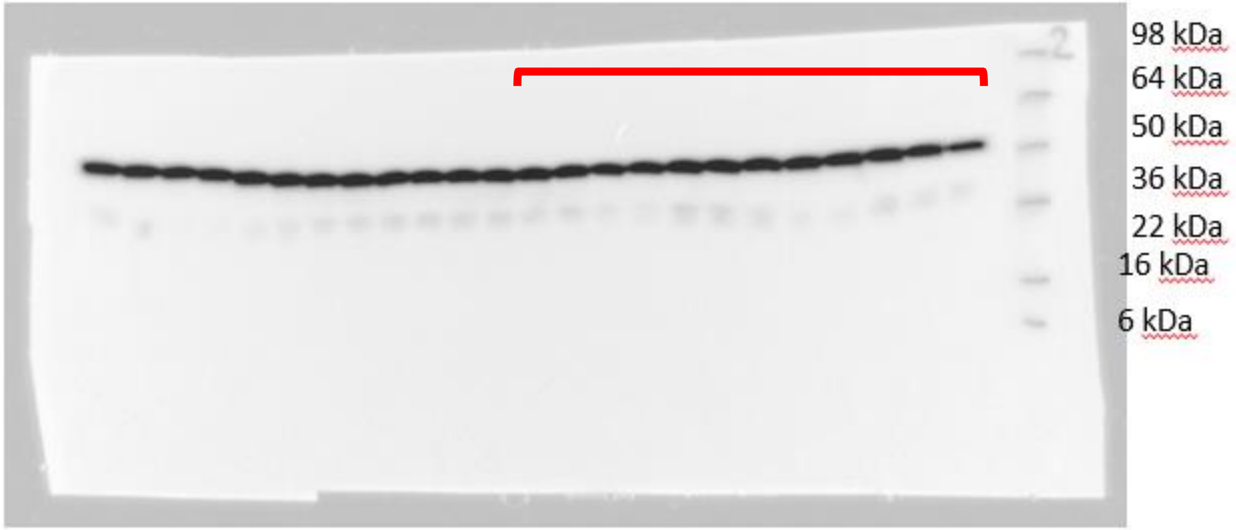

α Pgk1
Raw image

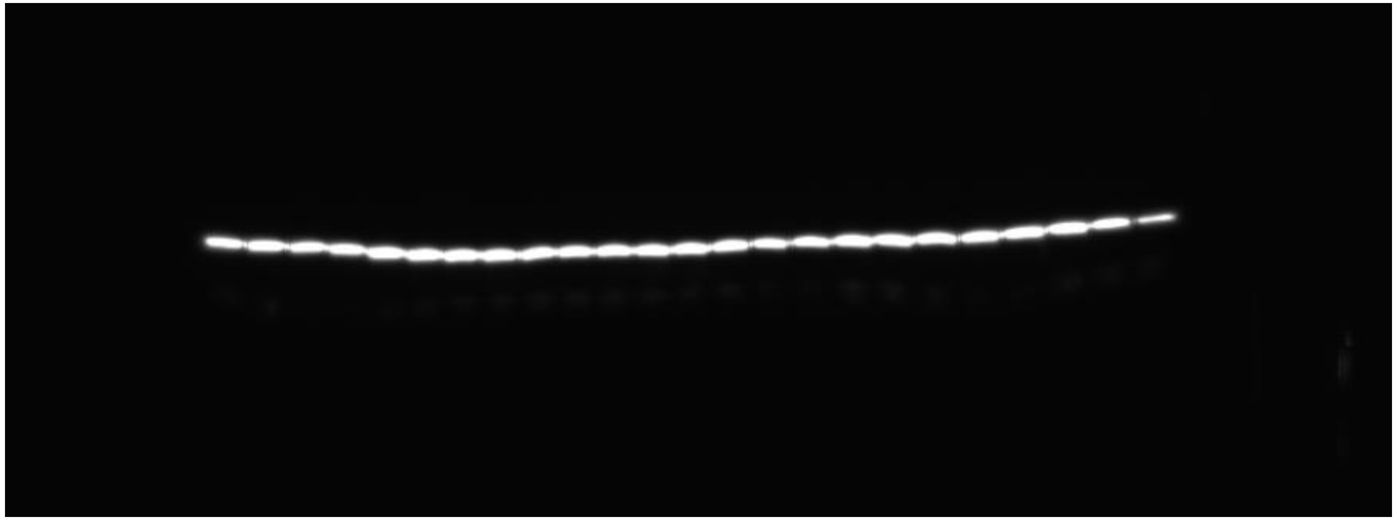

Figure 2A, top left western

α GST
overlaid with size marker

α GST
Raw image

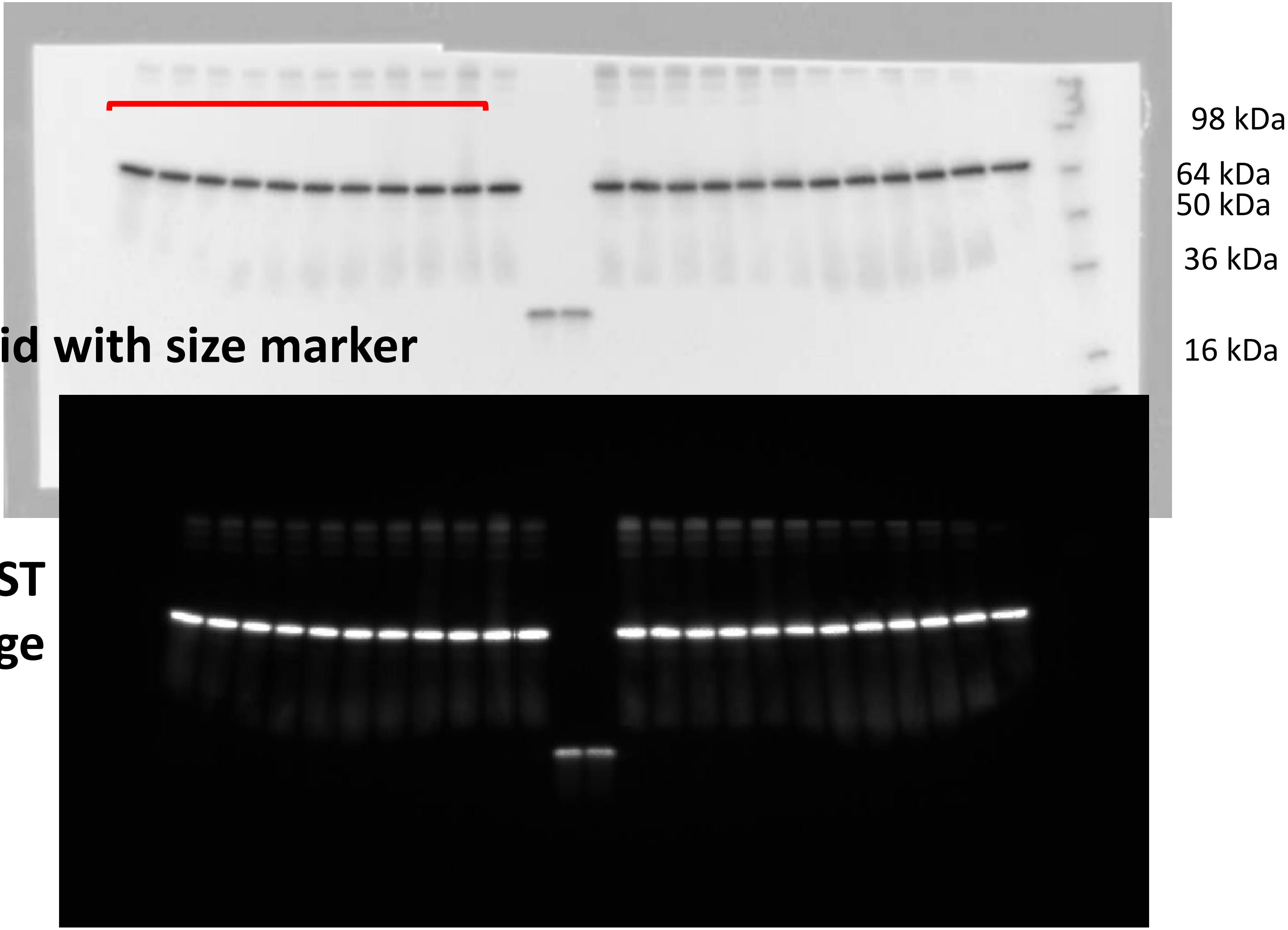

Figure 2A, top right western

α Pgk1
overlaid with size marker

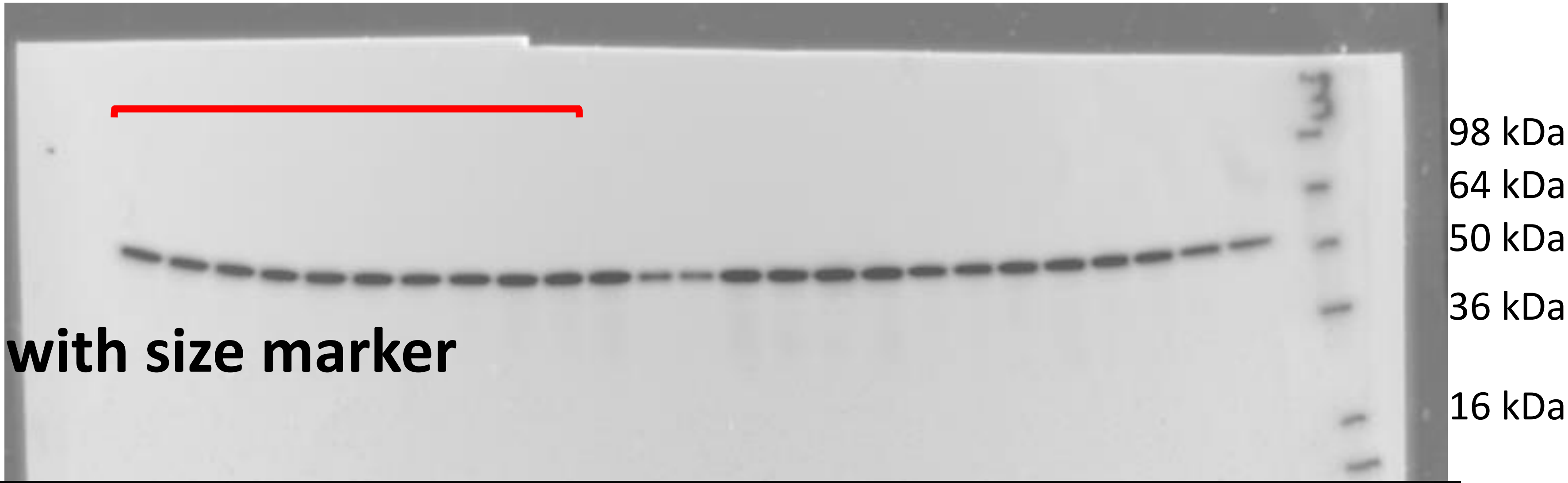

α Pgk1
Raw image

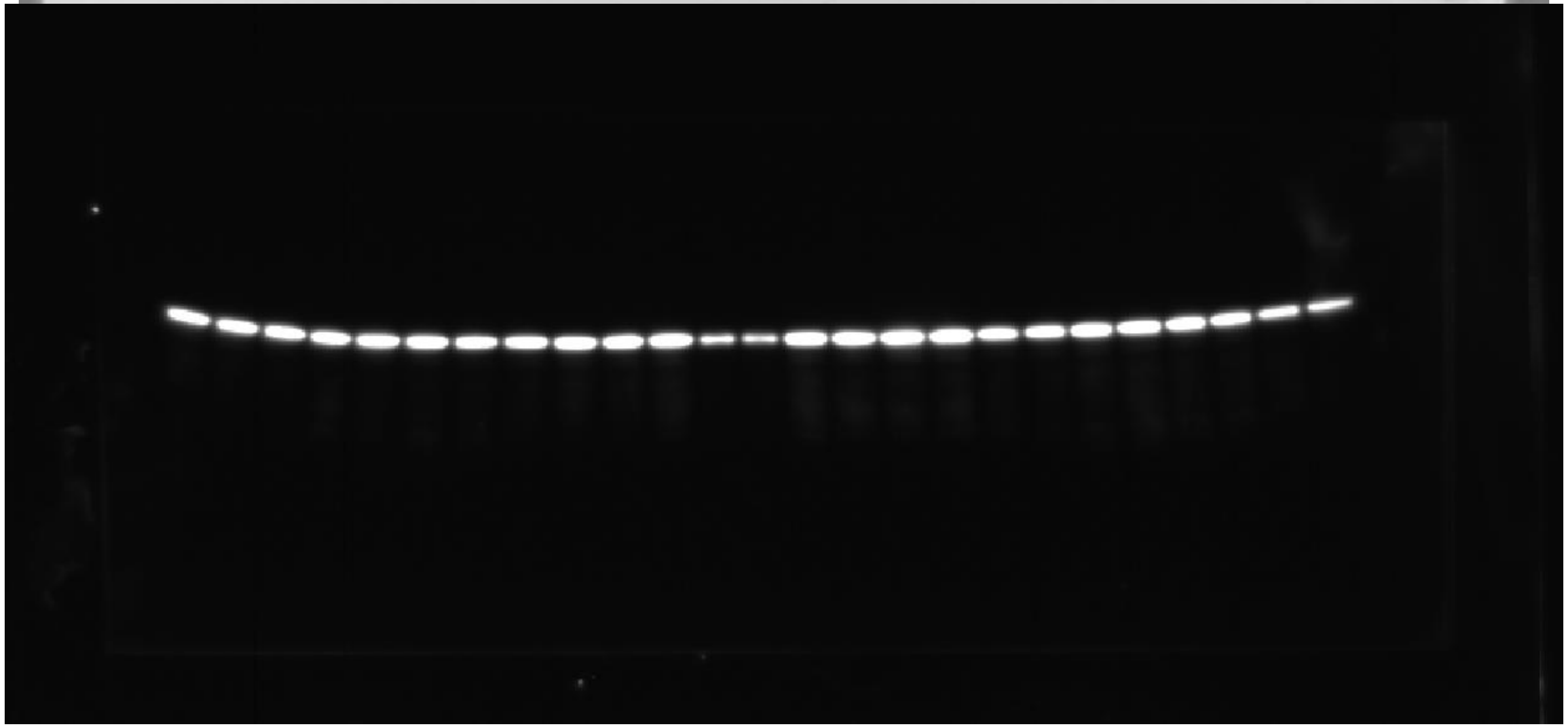

Figure 2A, top right western

α GST
overlaid with size marker
Mirror image shown in Figure 2A

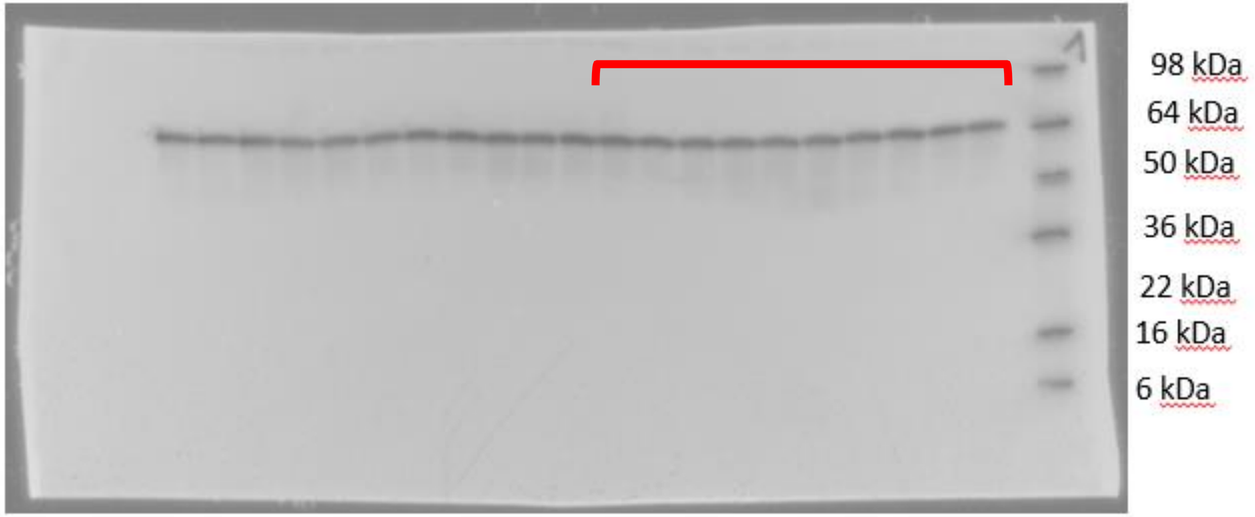

α GST
Raw image

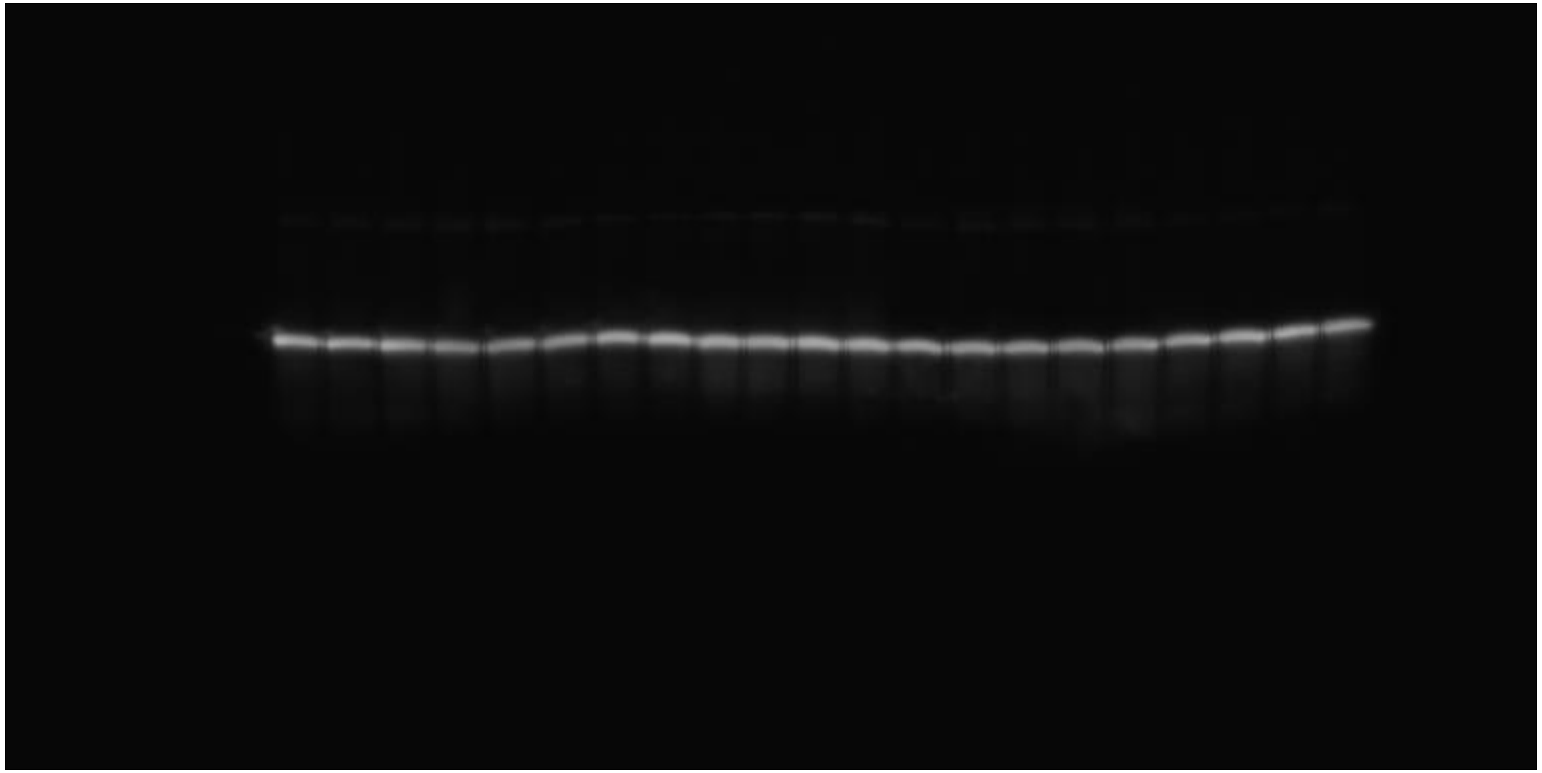

Figure 2A, middle left western

α Pgk1

overlaid with size marker

Mirror image shown in Figure 2A

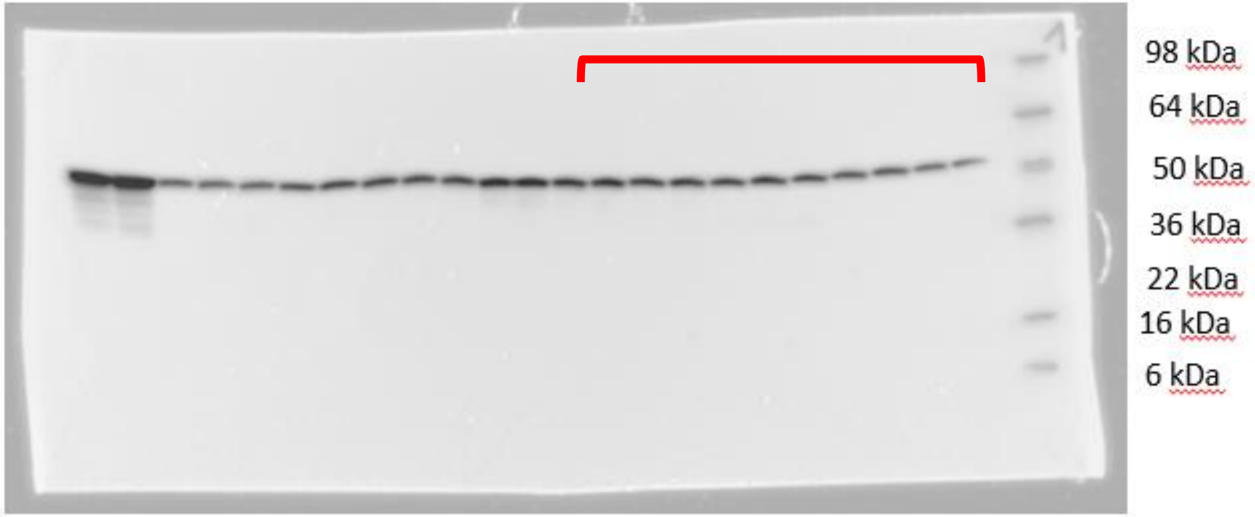

α Pgk1

Raw image

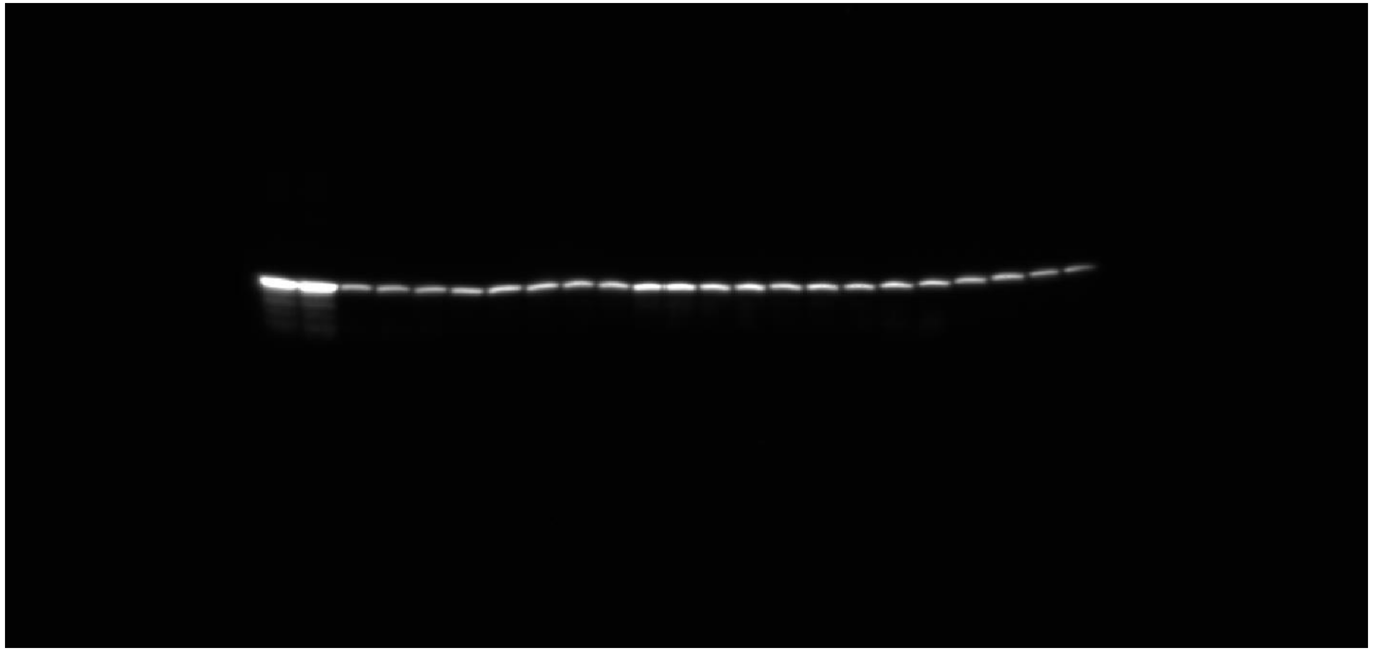

Figure 2A, middle left western

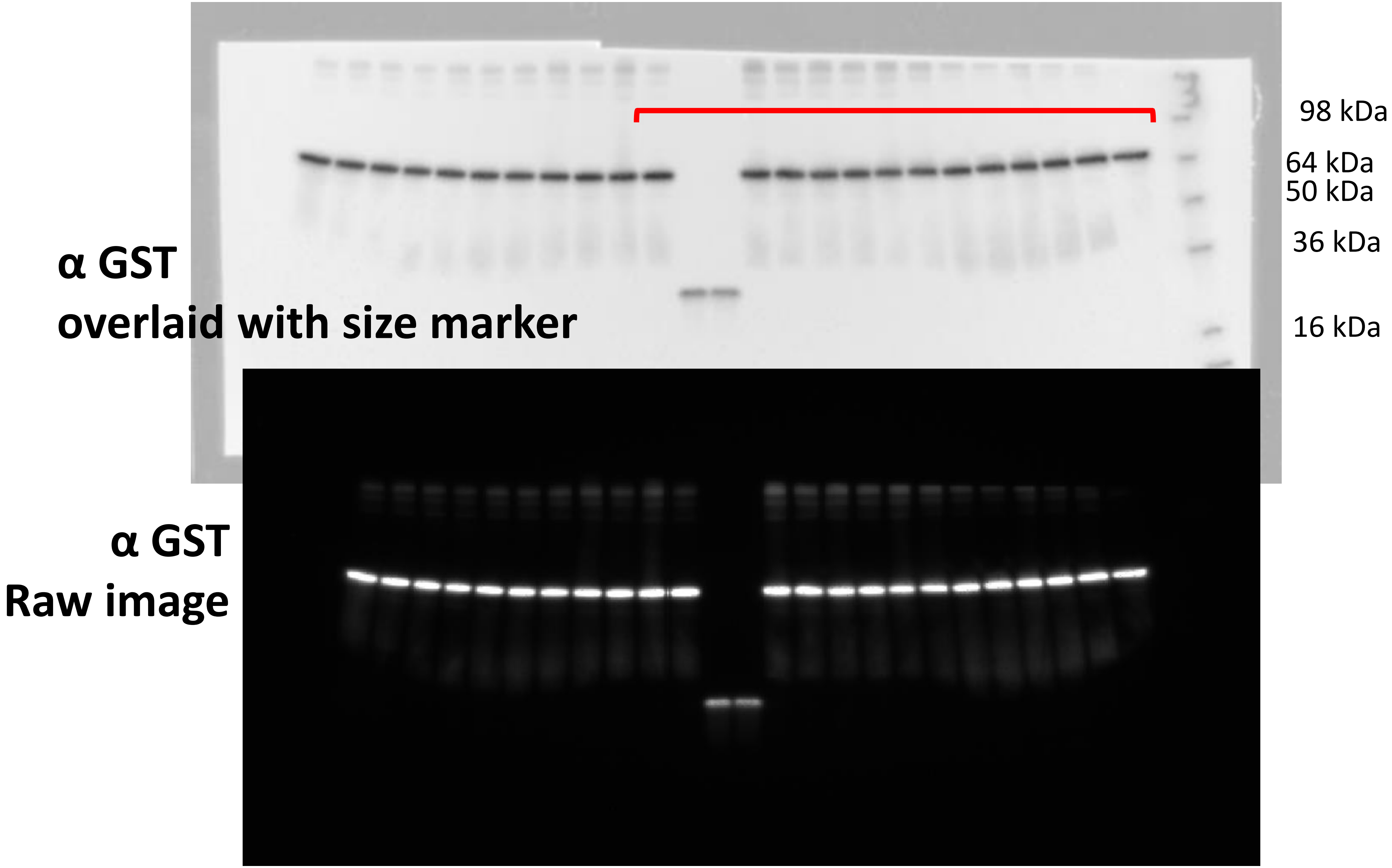

Figure 2A, middle right western

α Pgk1
overlaid with size marker

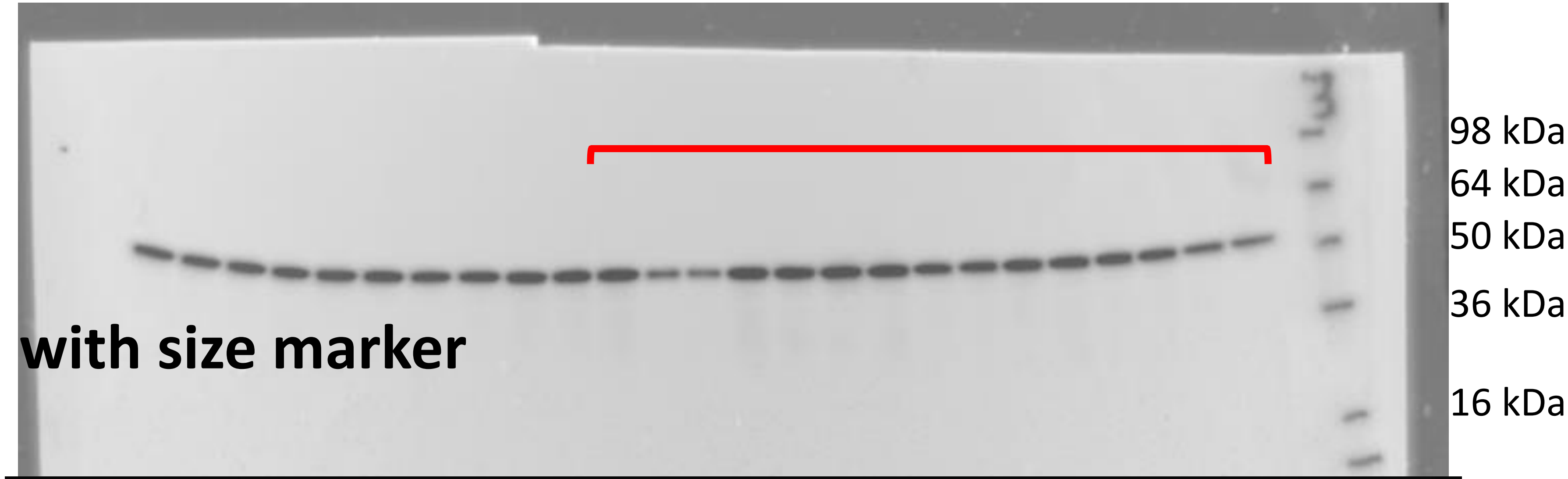

98 kDa
64 kDa
50 kDa
36 kDa
16 kDa

α Pgk1
Raw image

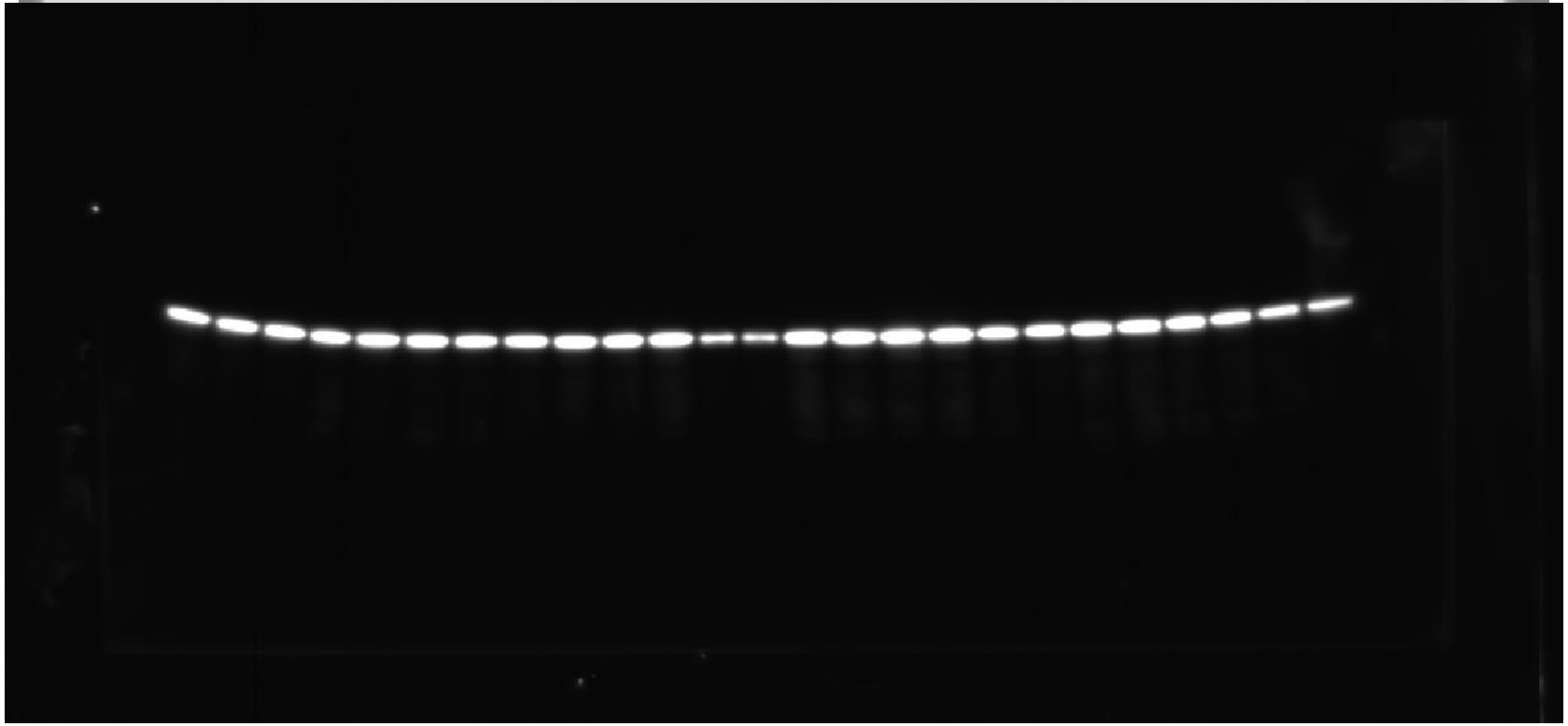

Figure 2A, middle right western

α GST

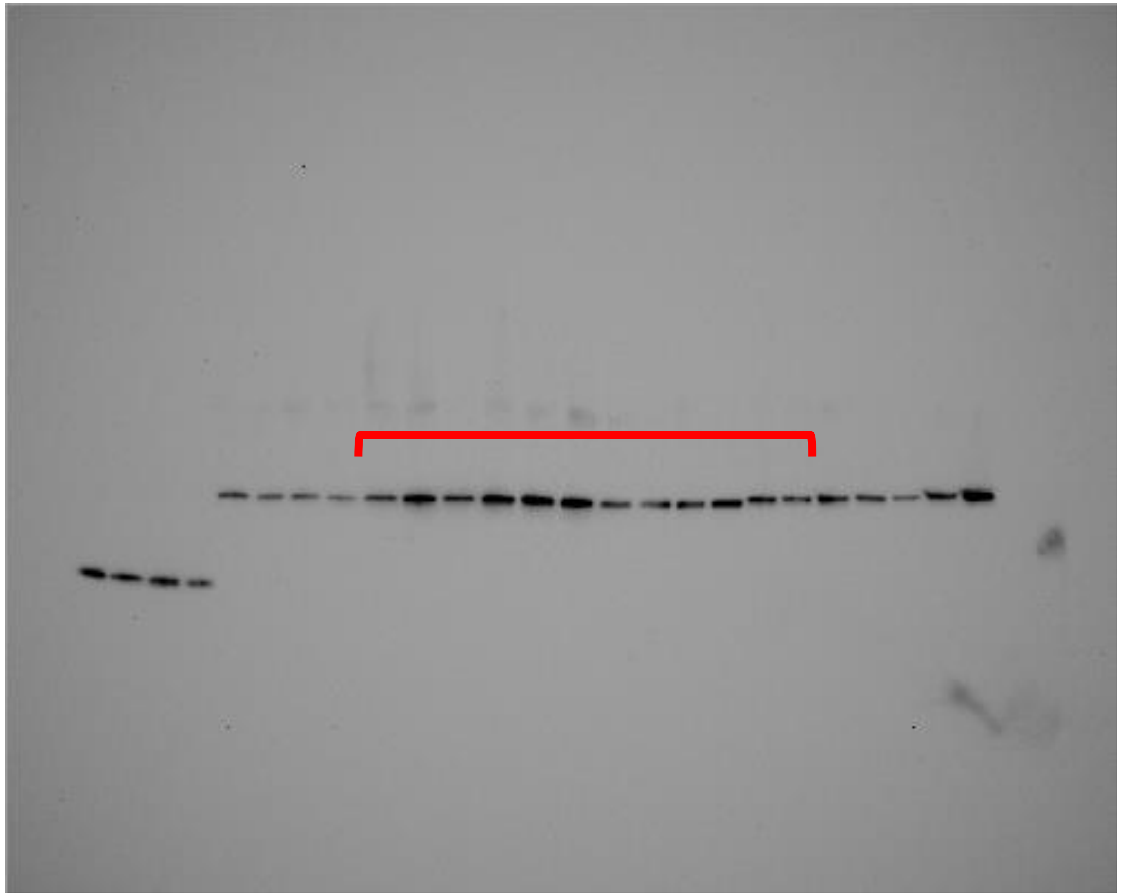

α Pgk1

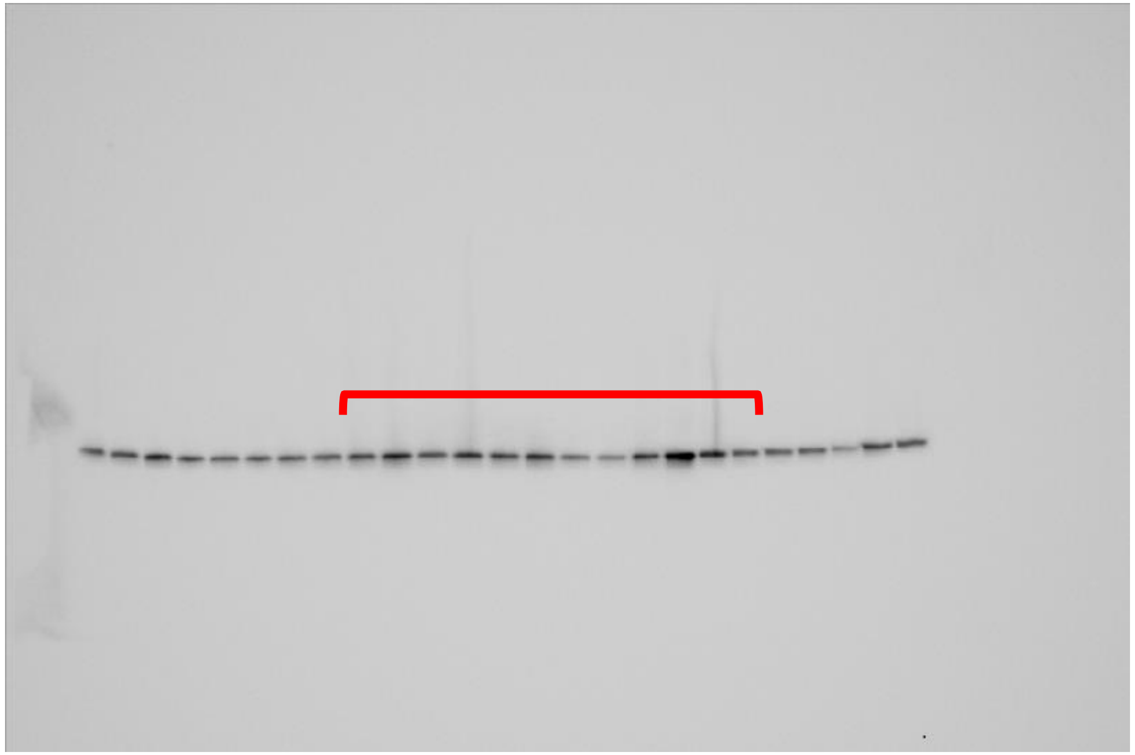

Figure 2A, bottom left western

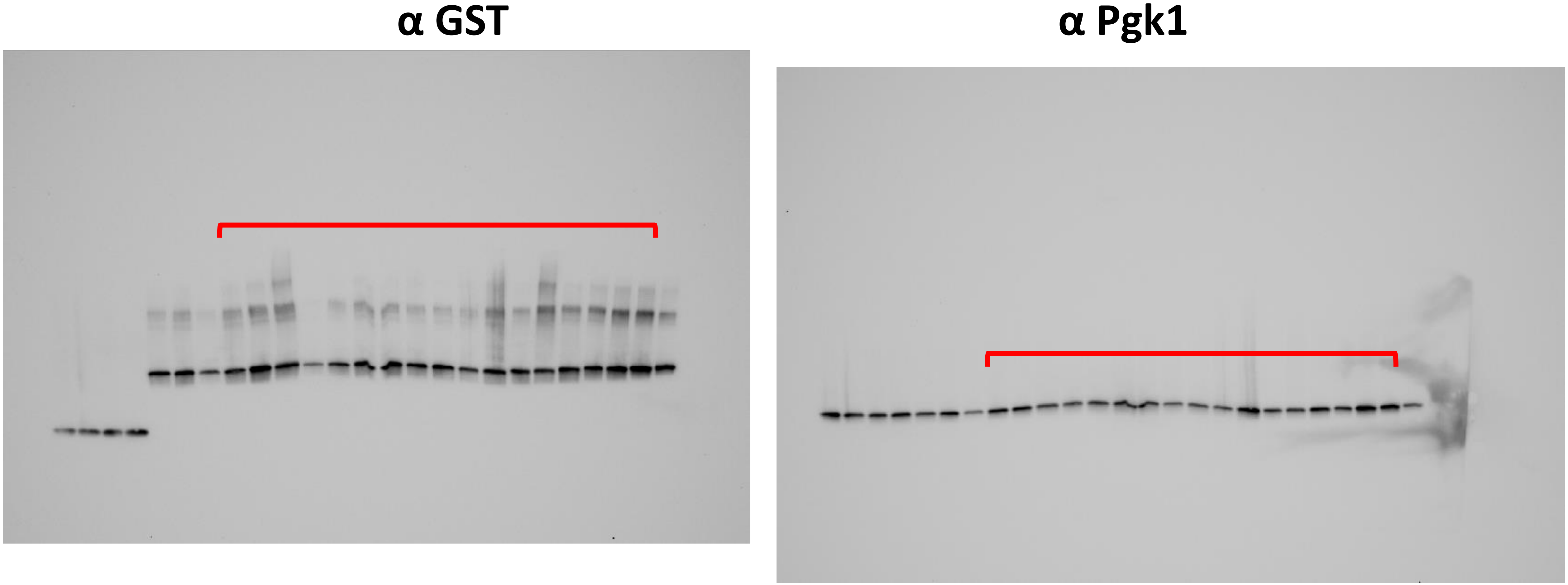

Figure 2A, bottom right western

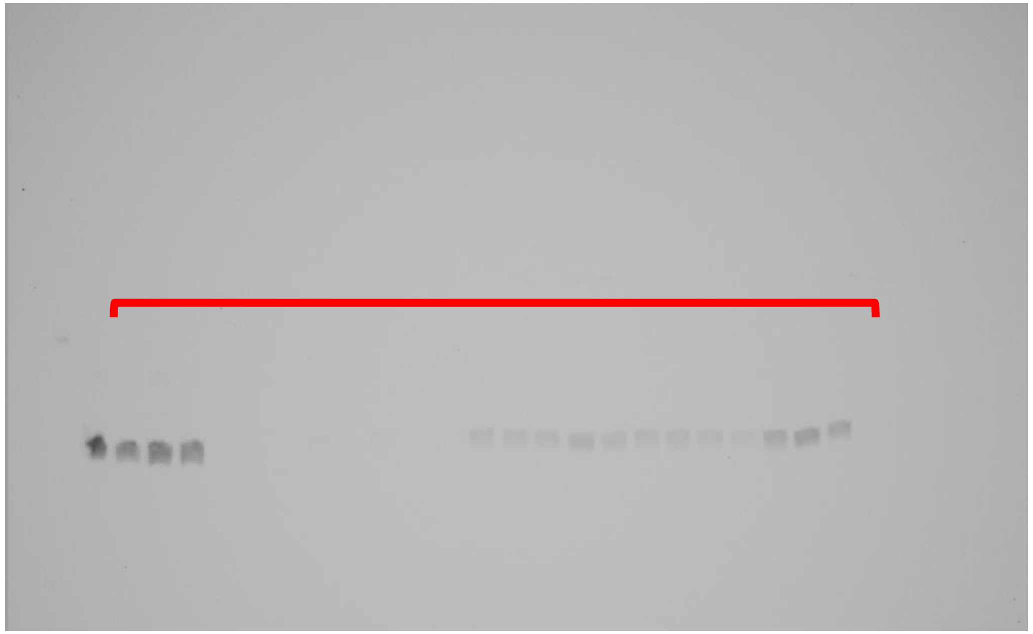

α eIF2α-P

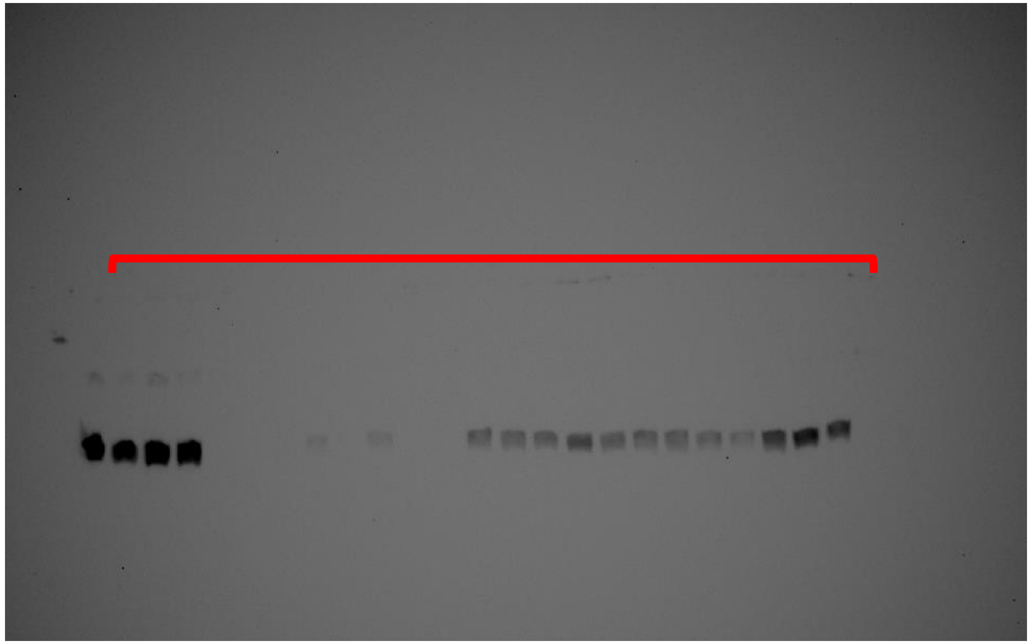

α eIF2α-P

α Pgk1

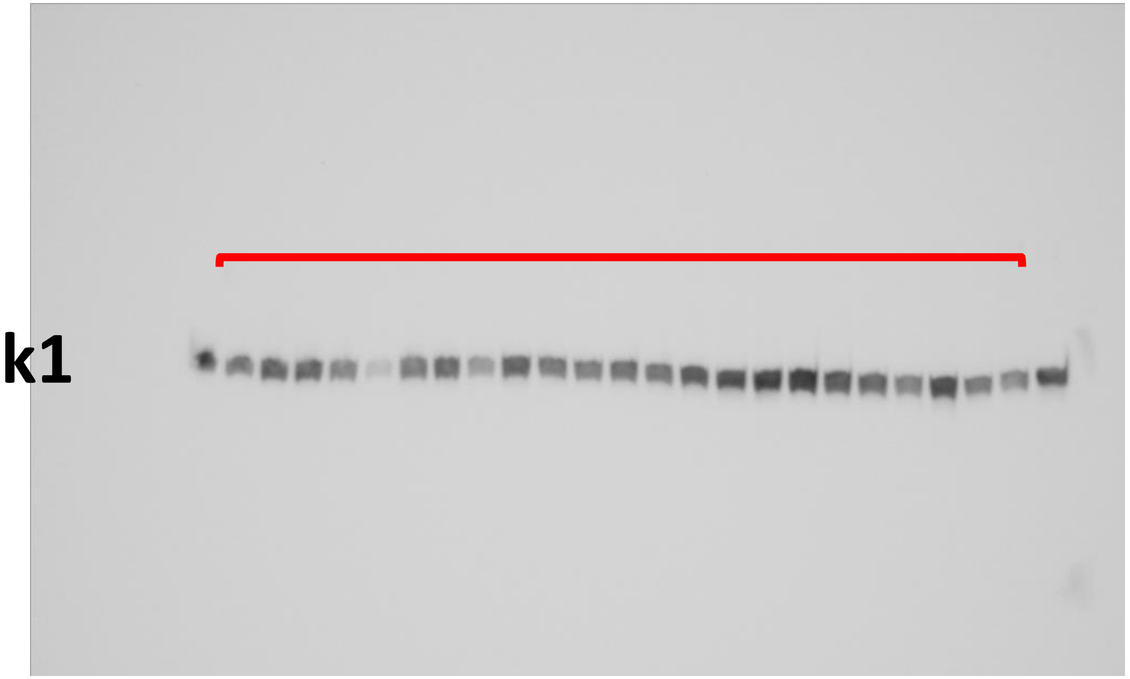

Figure 3A, left western

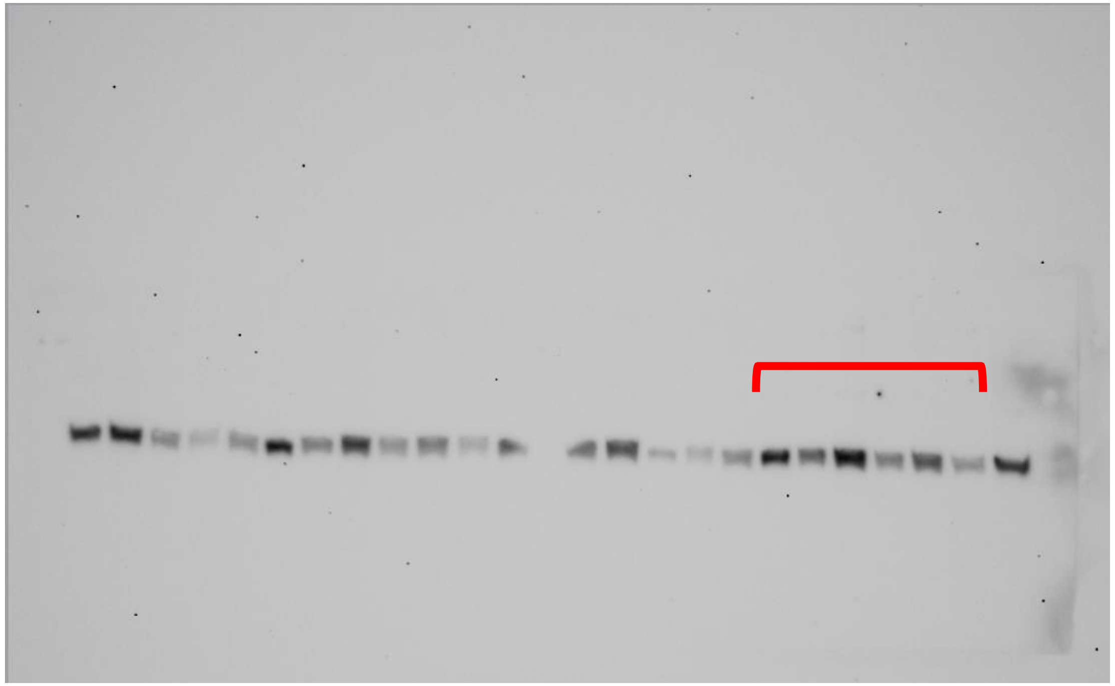

α eIF2α-P

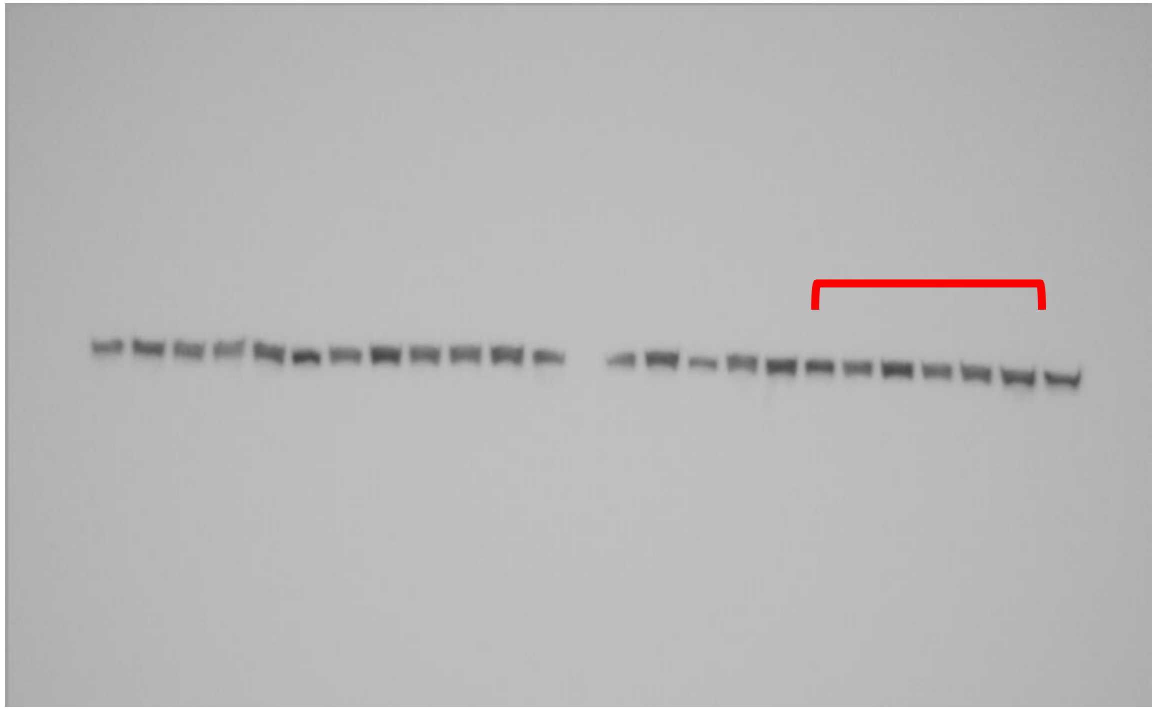

α Pgk1

Figure 3A, right western

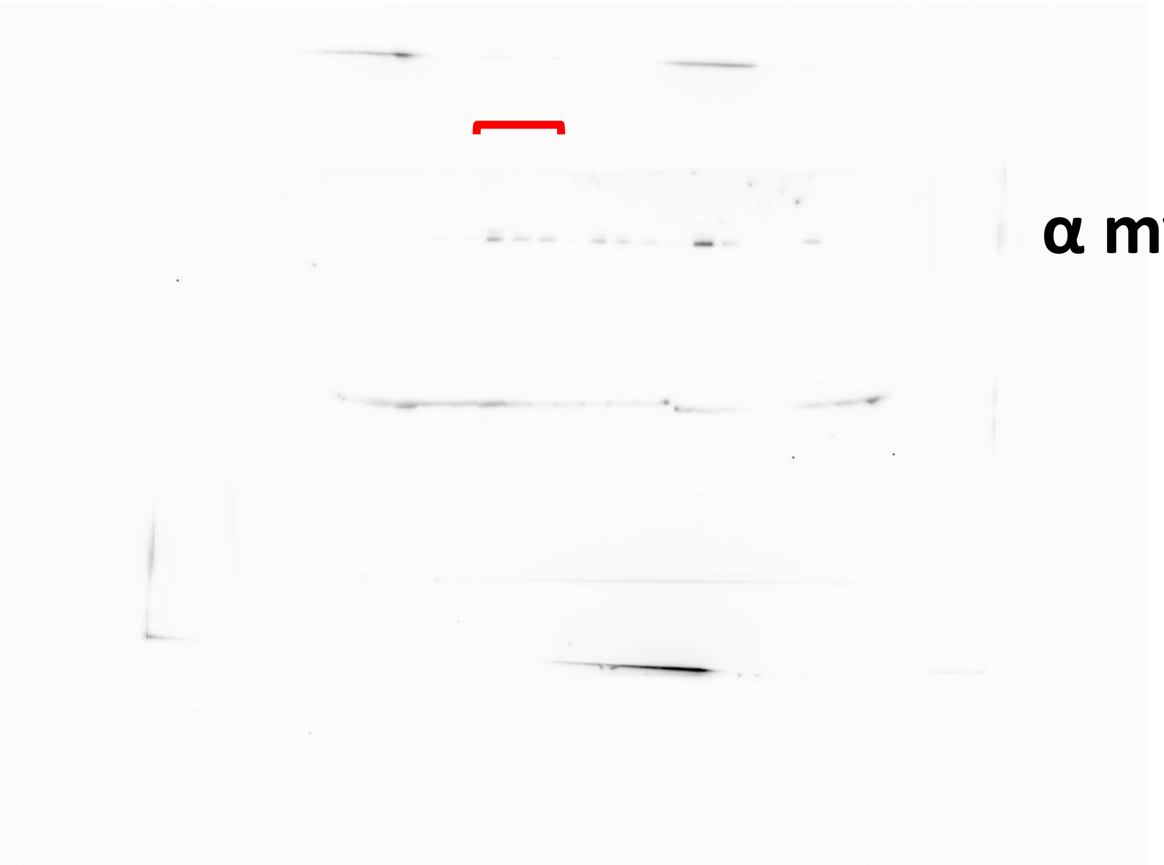

α myc

α Pgk1

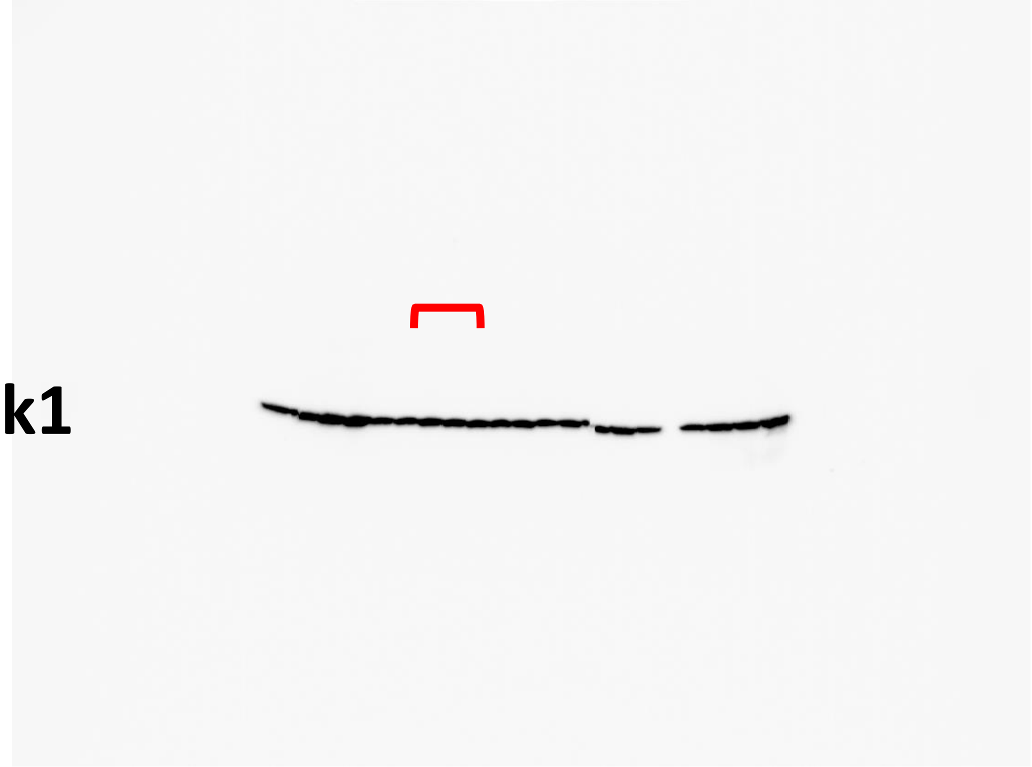

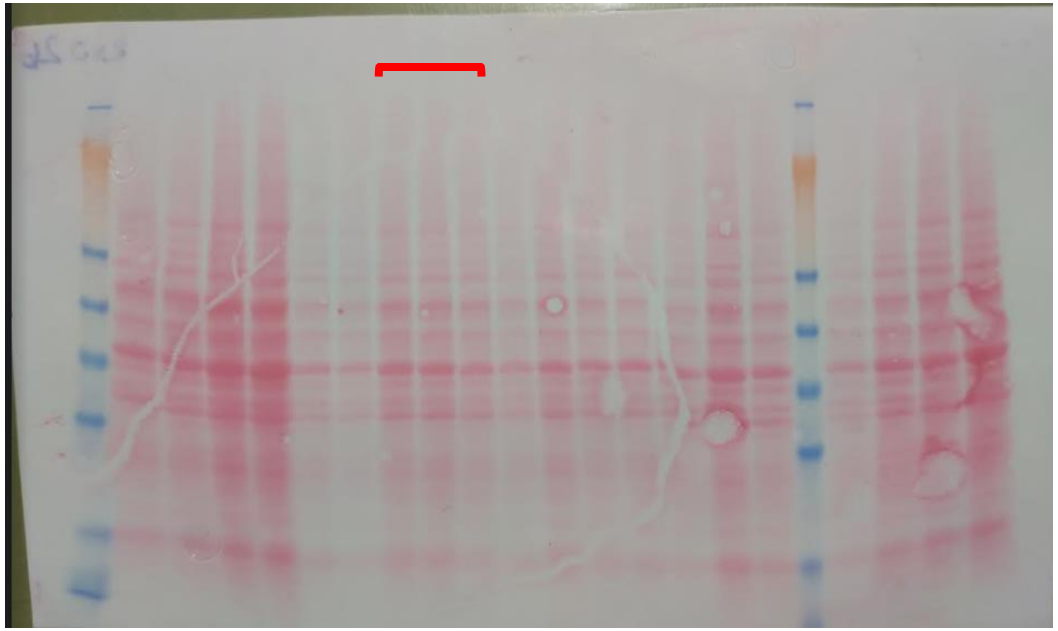

Figure 4B

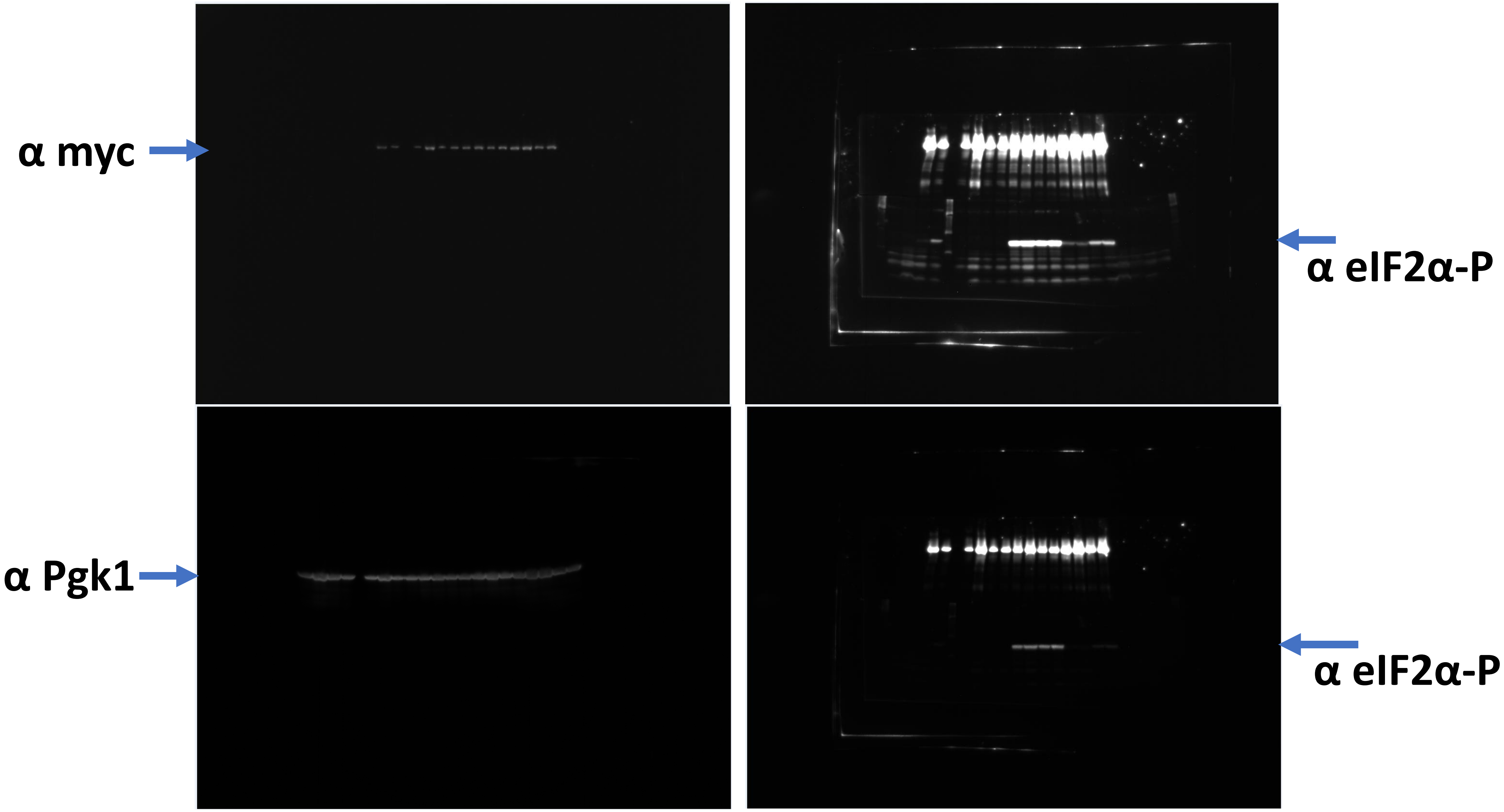

Figure 4C

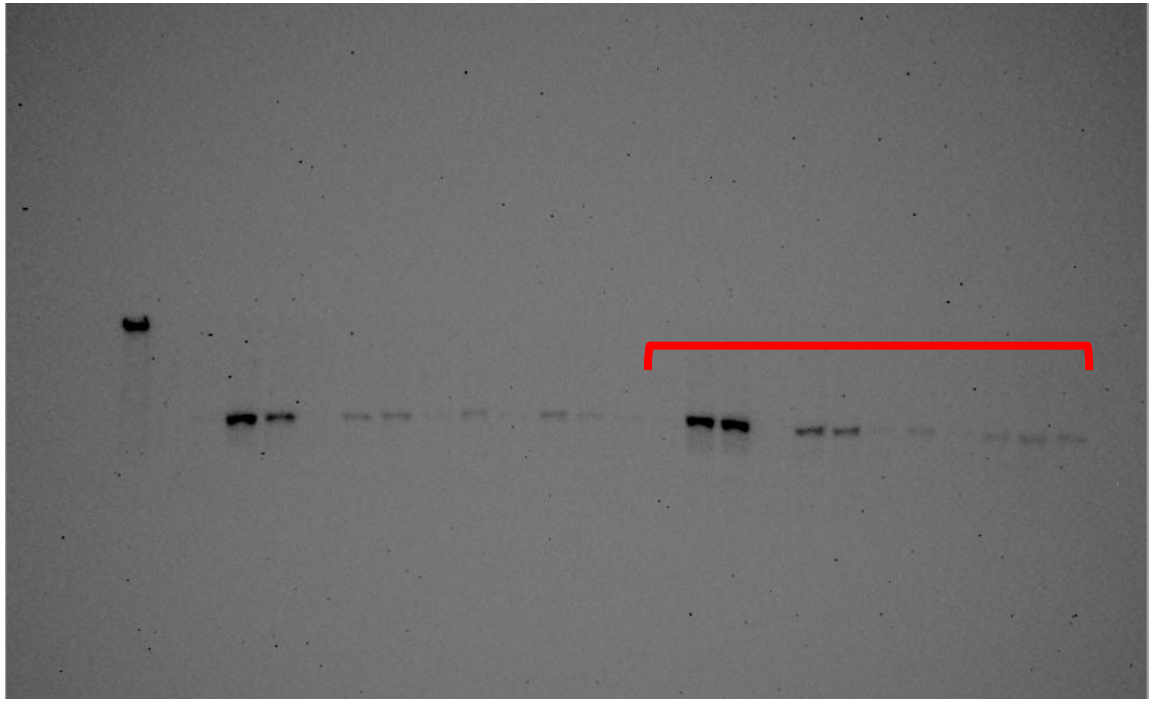

α eIF2α-P

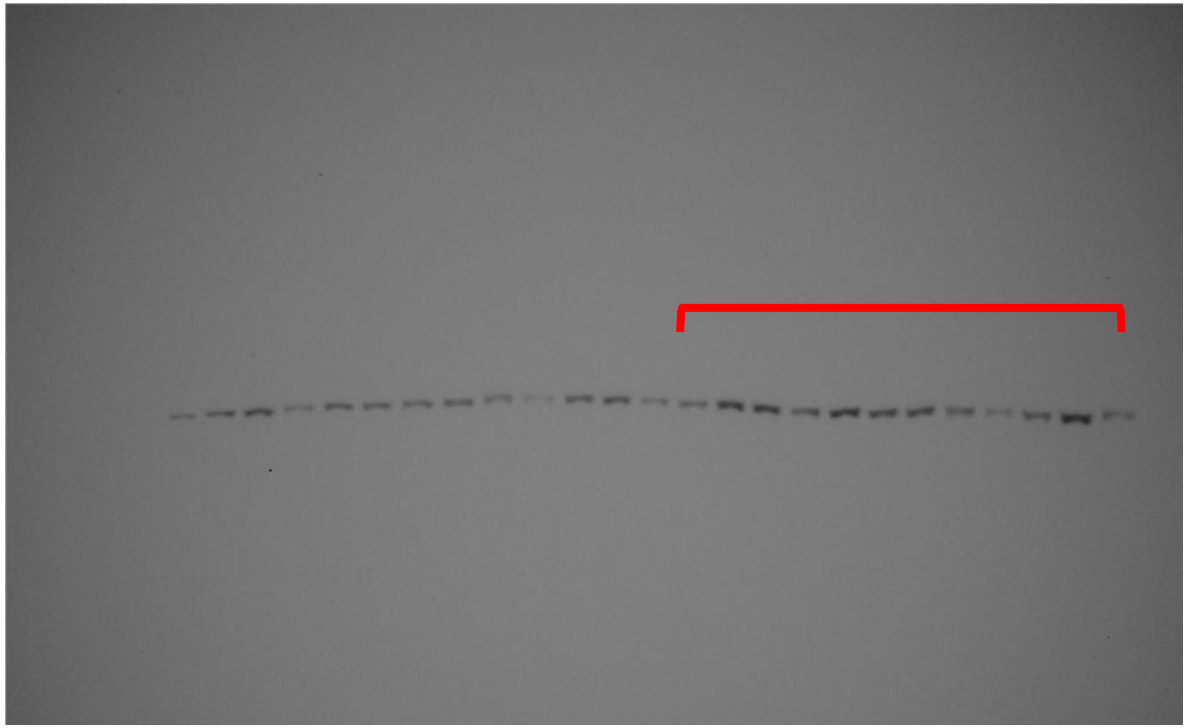

α Pgk1

Figure 5A
